# Economic evaluation of COVID-19 rapid antigen screening programs in the workplace

**DOI:** 10.1186/s12916-022-02641-5

**Published:** 2022-11-23

**Authors:** Thomas N. Vilches, Ellen Rafferty, Chad R. Wells, Alison P. Galvani, Seyed M. Moghadas

**Affiliations:** 1 Agent-Based Modelling Laboratory, York University, Toronto, Ontario Canada; 2 Institute of Health Economics, Edmonton, Alberta Canada; 3 Center for Infectious Disease Modeling and Analysis, Yale School of Public Health, New Haven, CT USA

**Keywords:** COVID-19, Rapid antigen Test, Screening, Simulations

## Abstract

**Background:**

Diagnostic testing has been pivotal in detecting SARS-CoV-2 infections and reducing transmission through the isolation of positive cases. We quantified the value of implementing frequent, rapid antigen (RA) testing in the workplace to identify screening programs that are cost-effective.

**Methods:**

To project the number of cases, hospitalizations, and deaths under alternative screening programs, we adapted an agent-based model of COVID-19 transmission and parameterized it with the demographics of Ontario, Canada, incorporating vaccination and waning of immunity. Taking into account healthcare costs and productivity losses associated with each program, we calculated the incremental cost-effectiveness ratio (ICER) with quality-adjusted life year (QALY) as the measure of effect. Considering RT-PCR testing of only severe cases as the baseline scenario, we estimated the incremental net monetary benefits (iNMB) of the screening programs with varying durations and initiation times, as well as different booster coverages of working adults.

**Results:**

Assuming a willingness-to-pay threshold of CDN$30,000 per QALY loss averted, twice weekly workplace screening was cost-effective only if the program started early during a surge. In most scenarios, the iNMB of RA screening without a confirmatory RT-PCR or RA test was comparable or higher than the iNMB for programs with a confirmatory test for RA-positive cases. When the program started early with a duration of at least 16 weeks and no confirmatory testing, the iNMB exceeded CDN$1.1 million per 100,000 population. Increasing booster coverage of working adults improved the iNMB of RA screening.

**Conclusions:**

Our findings indicate that frequent RA testing starting very early in a surge, without a confirmatory test, is a preferred screening program for the detection of asymptomatic infections in workplaces.

**Supplementary Information:**

The online version contains supplementary material available at 10.1186/s12916-022-02641-5.

## Background

Diagnostic testing has been instrumental to mitigating the COVID-19 pandemic, particularly for informing quarantine strategies, and evaluating spatiotemporal infection risk [1–5]. Prior to the widespread availability of rapid antigen (RA) tests, identifying SARS-CoV-2 infection relied predominantly on reverse transcription polymerase chain reaction (RT-PCR). The availability of RA tests has provided a viable alternative to RT-PCR methods by scaling up testing capacities and shortening test turnaround times from days to minutes [2, 6]. Despite their lower sensitivity compared to RT-PCR tests, low-cost self-administered RA tests are increasingly used outside clinical settings, especially for screening at home and workplaces [7, 8].

The effectiveness of RA tests in real-world settings has been demonstrated in several studies [8–14]. Relative to the detection of cases through RT-PCR screening for asymptomatic cases, RA tests have been able to identify 20–81% of these cases [2, 8, 12–17]. However, the fast turnaround time of RA tests can allow for increased frequency of testing compared to RT-PCR, thus improving case detection in the early stages of disease for screening programs and limiting the extent of onward transmission [17, 18].

A recent study provides a roadmap for the scalable implementation of frequent RA testing to detect asymptomatic infection in workplaces, suggesting that screening programs could interrupt chains of transmission, thereby reducing the burden of disease [10]. Although low rates of false positives in large-scale screening programs with frequent RA testing may not disrupt workplace operations [10], the scale of false-negative outcomes remains a concern, especially for the identification of breakthrough infections [8]. Commissioned by Health Canada, we evaluated the costs and benefits of frequent RA testing in workplaces post-Omicron BA.1 wave by performing cost-effectiveness analyses of screening programs with and without confirmatory RT-PCR testing.

## Methods

### General framework

To evaluate the cost-effectiveness of RA screening in workplaces, we used both direct and indirect costs associated with SARS-CoV-2 infection and outcomes derived from an agent-based model of COVID-19 transmission dynamics based on 500 Monte-Carlo replications (Additional file 1) [19–46]. We have previously used this model for estimating the impact of non-pharmaceutical interventions and vaccination on reducing the COVID-19 burden [19–21, 47]. Taking the province of Ontario, Canada as the population study, we simulated incidence of infections and outcomes over a 1-year time horizon from the beginning of April 2022. We accounted for the population immunity generated by vaccination in different age groups. The model was initiated with a 10% naturally acquired population immunity against infection and calibrated to an effective reproduction number of 1.2 [25]. The 10% proportion of the population with immunity due to a prior infection is based on the reported incidence [48], but it may be conservative given the possibility of undocumented asymptomatic or mild symptomatic infections.

We considered the primary and booster vaccination coverage in different age groups as of April 1, 2022 (status quo scenario) [49], accounting for the temporal waning of immunity post-vaccination or infection. Primary vaccination is defined as the first two doses of approved vaccines in Canada (i.e., Moderna SpikeVax™, or Pfizer-Bio-NTech Comirnaty). Booster refers to an additional (third) vaccine dose. Under the status quo, 81.2% of the Ontario population was fully vaccinated of whom 59% had received a booster. Among working adults aged 18–65 years, the coverage of booster vaccination was ~48% [50]. We also considered additional scenarios in which the booster coverage of working adults was increased by 20% and 80% over the status quo. Simulations for each testing scenario were run by implementing the model in Julia Language, and statistical analyses were conducted using outputs in MATLAB.

### Rapid antigen screening programs

For a given coverage of booster vaccination, we set the baseline scenario for the cost-effectiveness analysis to be “RT-PCR testing of only severe symptomatic cases” (TOSC) in the population. For the screening program, we used the distribution of workplace sizes in Ontario (Additional file 1: Fig. S2) [24]. Screening of asymptomatic infection was simulated as an incremental to the baseline for workplaces with at least 50 employees, and with testing every Monday and Thursday. We considered scenarios in which either 50% or 100% of workplaces participated in an RA screening program. As a requirement of the policy, we assumed that all individuals working in places with a screening program in effect adhere to the testing schedules. For the daily number of contacts inside and outside workplaces, we relied on recent empirical distributions from the CONNECT study on time trends in social contacts before and during the COVID-19 pandemic [23]. We assumed that the result of an RA test will be available within several minutes, but the result of an RT-PCR test will be available 1 day from sample collection.

To infer the temporal diagnostic sensitivity of the RT-PCR assay, we fitted a time-dependent log-Normal probability density function [17] to serial testing data [51], assuming that the maximum of this function coincides with the peak of infectiousness. The diagnostic sensitivity of the rapid antigen tests was then expressed as the product of the diagnostic sensitivity of the RT-PCR and the temporal percent positive agreement (PPA) of the rapid antigen tests with an RT-PCR test (Additional file 1) [2, 6, 51–57]. Although a number of RA tests have been used in Canada, we performed our analysis with temporal diagnostic sensitivity of Abbott-Panbio^TM^ (described in the Results section), as well as BD Veritor and Sofia tests (Additional file 1) derived based on the temporal PPA with an RT-PCR test relative to the time of symptom onset [54, 55]. A specificity of 99.8% was used for the RA tests and 99.9% for the RT-PCR test.

**Fig. 1:**
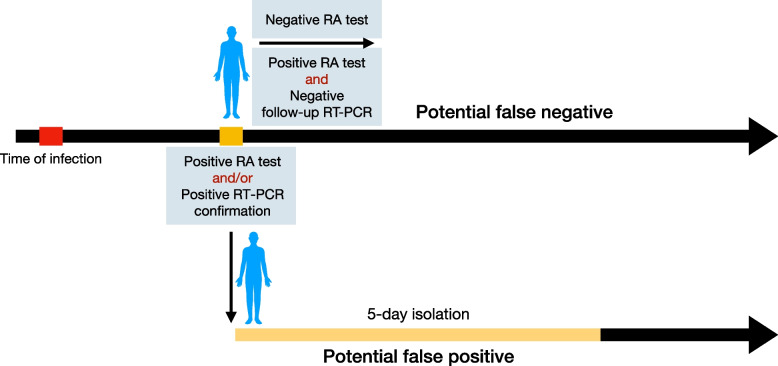
Schematic illustration of testing and possible outcomes with isolation after infection. For the screening program with a confirmatory RT-PCR testing (SP2), RA-positive cases are required to isolate while awaiting the RT-PCR test result

Screening programs were implemented with and without a confirmatory test for RA-positive cases (Table 1). For the screening program without a confirmatory test (SP1), if the RA test was positive, individuals would complete a 5-day isolation period before returning to work and normal activities (Fig. 1). For the RA screening program with either a confirmatory RT-PCR test (SP2) or a confirmatory RA test (SP3) 1 day after the initial RA positive [7, 58], the 5-day isolation period was reduced to 1 day if the confirmatory test was negative. The infectious period for an individual may be sampled to be longer than the isolation period (Additional file 1: Section 2). In this case, contacts will be associated with risk of disease transmission upon return to the workplace and normal activities.

**Table 1:** Rapid antigen screening programs evaluated and compared with the baseline of RT-PCR testing of only severe symptomatic cases. Screening programs SP1, SP2, and SP3 are incremental to the baseline and include testing of severe cases

Scenarios	Target population	Status	Test (result)	Action 1	Action 2
Baseline	General population	Severe symptomatic	RT-PCR(+)	Start isolation for 5 days	None
**Screening program**					
SP1	Workplace	Asymptomatic	RA(+)	Start isolation for 5 days	None
SP2	Workplace	Asymptomatic	RA(+)	Start isolation for 5 days and follow up with an RT-PCR test on the same day of initial RA(+)	If RT-PCR+, complete 5 days of isolation, otherwise end isolation
SP3	Workplace	Asymptomatic	RA(+)	Start isolation for 5 days and follow up with RA test a day after initial RA(+)	If second RA+, complete 5 days of isolation, otherwise end isolation

The baseline of TOSC was implemented throughout the simulations. We then varied the initiation of RA screening at the workplace, considering programs with a duration of 16, 32, and 52 weeks. Comparison between RA screening programs and TOSC was done at the same coverage of primary and booster vaccination.

### Cost-effectiveness analysis

We conducted a cost-effectiveness analysis of the RA screening programs (Table 1), with the benefits captured as QALYs. To estimate total costs and benefits, we used the number of mild and severe symptomatic infections, outpatient and emergency department visits, hospitalizations and ICU admissions, isolation days after a positive test and a false positive RA test, deaths, and the total number of different tests performed. Costs were captured from two perspectives of (i) healthcare, which included those associated with health outcomes and testing, and (ii) productivity loss due to illness (i.e., isolation for acute infection, hospitalization, and death), as well as time lost to testing (Table 2). All costs were converted and inflated to Canadian dollars in 2021 [72].

**Table 2:** Costs (in 2021 Canadian dollars) and effects associated with disease outcomes and testing programs used in cost-effectiveness analysis. Calculation for long-COVID and productivity loss associated with premature deaths are detailed in Additional file 2

Input parameter	Description	Cost estimate (CAD$)	Source
**Testing (direct)**			
RA testing	Procurement and shipping of RA testing	8.00	Health Canada communication
RT-PCR testing	Testing includes human resource and laboratory capacity requirements	60.67	[59]
**Health care costs**			
Emergency room	Per positive case	22.00	[60]
Outpatient	Per symptomatic case	6.45	[61, 62]
Non-ICU hospitalization	Average per patient	12,587.76	[63]
ICU hospitalization	Average per patient	52,756.06	[63]
Chronic COVID-19 symptoms per hospitalized case^a,b^	Net present cost of chronic COVID-19 symptoms	20,870.714	[64, 65]
**Productivity loss by age group (indirect)**			
RA testing	To perform an RA test and obtain results	0.00	
RT-PCR test with a 0.5-h testing time	One-day isolation to receive results of confirmatory test	Under 15 yrs: 0.00 15–24 yrs: 56.98 25–34 yrs: 187.12 35–44 yrs: 245.27 45–54 yrs: 251.24 55–64 yrs: 87.86 65+ yrs: 66.82	[66–68]
Isolation of positive cases	5-day isolation as recommended by public health in Canada	Under 15 yrs: 0.00 15–24 yrs: 267.09 25–34 yrs: 877.14 35–44 yrs: 1,149.72 45–54 yrs: 1,177.68 55–64 yrs: 411.84 65+ yrs: 313.23	[67, 68]
Hospitalization	Loss of work productivity during hospitalization and recovery from illness	Under 15 yrs: 0.00 15–24 yrs: 2,093.96 25–34 yrs: 6,876.80 35–44 yrs: 9,013.81 45–54 yrs: 9,232.99 55–64 yrs: 3,228.82 65+ yrs: 2,455.72	[63, 65, 67, 68]
Death due to COVID-19^b^	Net present loss of work productivity from premature mortality	Under 15 yrs: 2,317,833.40 15–24 yrs: 2,092,194.38 25–34 yrs: 1,870,791.62 35–44 yrs: 1,613,844.69 45–54 yrs: 1,315,647.28 55–64 yrs: 969,577.01 65+ yrs: 776,233.85	[67, 68]
**Measure of effect**			
QALY decrement per symptomatic case	Calculated during acute symptomatic infection	0.009	[69]
QALY decrement per hospitalized case^a,b^	Calculated during hospitalization and recovery	0.44	[70]
QALY decrement per death^b^	Net present calculated based on life expectancy at the age of death	Under 15 yrs: 39.31 15–24 yrs: 35.48 25–34 yrs: 31.73 35–44 yrs: 27.37 45–54 yrs: 22.31 55–65 yrs: 16.44 65+ yrs: 13.16	[71]

*RA* rapid antigen, *RT-PCR* reverse transcription polymerase chain reaction, *yrs* years, *QALY* quality-adjusted life year

^a^Based on a time horizon of medical costs of 5 years

^b^Discounted at a rate of 1.5%

Cost-effectiveness results are presented by both the incremental cost-effectiveness ratio (ICER), and the incremental net monetary benefits (iNMB) for direct comparison of the testing scenarios. An ICER was calculated for each testing scenario, in comparison to the baseline of TOSC. NMB was calculated by subtracting the costs of a scenario from the monetary value of health gained using a willingness-to-pay (WTP) threshold of $CAD 30,000 [73].

## Results

### Effectiveness of testing programs

**Fig. 2:**
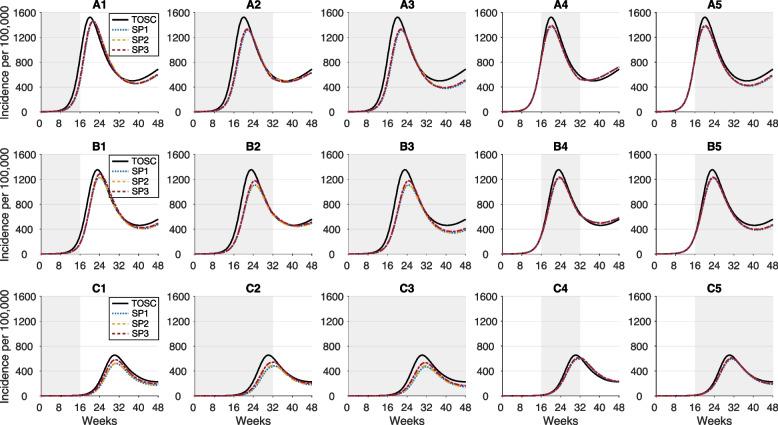
Projected average daily incidence of all (symptomatic and asymptomatic) infections for TOSC (black); SP1 in workplaces without a confirmatory test (blue); SP2 in workplaces with a confirmatory RT-PCR test (orange); and SP3 in workplaces with a confirmatory RA test (red). Screening programs were implemented for 50% of workplaces with a size of 50+ employees. The booster vaccination among adults aged 18–64 years was set to reported coverage as of April 1, 2022 [status quo] (**A1**–**A5**); an increase of 20% over status quo (**B1**–**B5**); and an increase of 80% over status quo (**C1**–**C5**). Shaded areas indicate the duration of SP1, SP2, and SP3; testing of severe cases with RT-PCR tests was implemented throughout the entire simulation. For SP2, RA-positive cases were isolated while awaiting the RT-PCR test result

Compared to TOSC in the general population, the largest reduction of cumulative incidence was achieved when the workplace screening programs were implemented for the entire year without interruption (Fig. 2A3, B3, and C3). For screening programs with a shorter duration, an earlier start during a surge resulted in a lower cumulative incidence. For example, RA screening that initiated at the start of a surge for a duration of 16 weeks reduced the total incidence (compared with TOSC) by 9.84% (95% credible interval [CrI]: 6.78% to 13.04%) in SP1, 7.97% (95% CrI: 5.57% to 11.4%) in SP2, and 9.28 (95% CrI: 5.47 to 12.35) in SP3 (Fig. 2A1). With the same duration of RA screening but delaying the program until 16 weeks after the start of the surge (Fig. 2A4), the reduction of incidence was 2.81% (95% CrI: 0.65% to 5.59%) in SP1, 2.77% (95% CrI: 0.52% to 4.51%) in SP2, and 2.43 (95% CrI: 0.29 to 4.44) in SP3. These outcomes were qualitatively independent of the proportion of workplaces that participated in the RA screening programs, or the coverage of booster vaccination (Fig. 2; Additional file 1: Fig. S5). However, increasing booster coverage of working adults had a significant effect on both delaying and suppressing the surge.

### Cost-effectiveness of testing programs (status quo scenario)

**Fig. 3:**
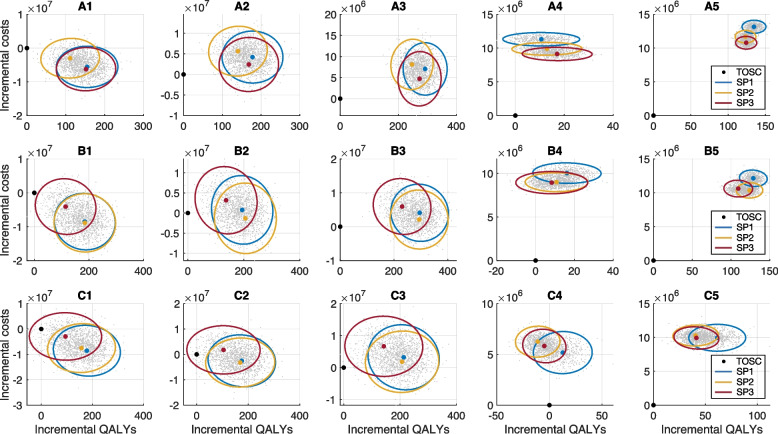
Cost-effectiveness plane derived from 500 independent Monte-Carlo simulations for different testing scenarios with the associated 95% credible ellipse of the data point distributions. Colors correspond to testing only severe cases (black dot); SP1 in workplaces without a confirmatory test (blue); SP2 in workplaces with a confirmatory RT-PCR test (orange); and SP3 in workplaces with a confirmatory RA test (red). Screening programs were implemented for 50% of workplaces with a size of 50+ employees. The booster vaccination among adults aged 18–64 years was set to reported coverage as of April 1, 2022 [status quo] (**A1**−**A5**); an increase of 20% over status quo (**B1**–**B5**); and an increase of 80% over status quo (**C1**–**C5**). Comparison was done between the baseline for testing only severe cases (TOSC) and each of the screening programs with the same booster coverage. Costs are in 2021 Canadian dollars

When RA screening was implemented by 50% of workplaces, SP1 with a 16-week duration from the start of a surge (Fig. 2A1) resulted in an average gain of 155 QALYs per 100,000 population with incremental costs of $−5,566,013, compared to the baseline of TOSC with status quo vaccine coverage. This produces the median ICER value of −35,739 (95% CrI: −83,556 to −5,375) per QALYs gained (Additional file 1: Table S5), suggesting that SP1 is a cost-saving (dominant) program (Fig. 3). We estimated $10.2 (95% CrI: 5.3, 15.7) million iNMB associated with this RA testing program (Table 3). For the same duration of the RA screening, SP2 generated an average gain of 111 QALYs per 100,000 population with incremental costs of $−3,001,675. The median ICER associated with SP2 was estimated at −27,828 (95% CrI: −93,085 to 16,388) per QALYs gained, with a 89% probability of being cost-saving; however, its iNMB was reduced, compared to SP1, to an estimated median of $6.4 (95% CrI: 0.9, 11.5) million. For RA testing with SP3, an average of 149 QALYs per 100,000 population was gained with incremental costs of $−6,215,098, resulting in the median ICER value of −42,166 (95% CrI: −95,002 to −7681) per QALYs gained. This suggests that SP3 is a cost-saving (dominant) program. The iNMB generated by SP3 was estimated to be $10.2 (95% CrI: 5.0 to 15.9) million.

**Table 3:** Estimated median iNMB (in million CDN$) and 95% credible intervals [CrI] of the mean iNMB values for comparing each testing program with the baseline scenario of testing only severe cases, and with timelines corresponding to scenarios in Fig. 2

Booster coverage	Duration of the screening program in weeks										Testing program
	0 to 16		0 to 32		0 to 52		16 to 32		16 to 52		
	iNMB	95% CrI	iNMB	95% CrI	iNMB	95% CrI	iNMB	95% CrI	iNMB	95% CrI	
Status quo as of April 1, 2022	10.2	5.3, 15.7	1.1	−4.0, 7.1	1.7	−3.3, 7.8	−11.0	−11.9, −10.2	−9.1	−10.0, −8.3	SP1
	6.4	0.9, 11.5	−1.5	−6.7, 4.0	−0.8	−6.1, 4.7	−9.5	−10.4, −8.7	−8.0	−8.7, −7.2	SP2
	10.2	5.0, 15.9	2.0	−3.0, 7.8	2.9	−2.2, 8.6	−8.6	−9.5, −7.7	−7.1	−7.9, −6.3	SP3
20% increase over status quo	14.3	6.4, 21.6	5.3	−2.9, 12.8	5.0	−3.0, 12.3	−9.5	−10.7, −8.5	−8.2	−9.4, −7.2	SP1
	14.1	6.4, 21.7	7.1	−0.5, 15.0	6.4	−1.5, 14.1	−8.7	−9.9, −7.7	−6.7	−7.9, −5.7	SP2
	7.1	−0.6, 13.9	0.4	−7.3, 7.7	0.4	−7.2, 7.6	−8.7	−10.0, −7.7	−7.3	−8.6, −6.4	SP3
80% increase over status quo	13.8	4.7, 22.1	7.7	−0.8, 16.0	3.0	−5.1, 11.2	−4.8	−6.6, −3.1	−8.2	−10.0, −6.4	SP1
	11.8	3.3, 20.4	7.7	−0.9, 16.7	3.7	−4.6, 12.8	−6.6	−8.0, −5.2	−9.1	−10.5, −7.8	SP2
	5.4	−3.3, 13.7	1.1	−7.3, 9.2	−2.7	−11.0, 5.2	−6.0	−7.4, −4.6	−8.8	−10.1, −7.3	SP3

As the duration of screening extended, the monetary benefits of RA testing reduced. For example, with a 32-week screening (Fig. 3A2), SP1 was cost-effective with a 62% probability (at the WTP threshold) with an estimated median ICER of 25,033 (95% CrI: -4,121 to 55,925). However, producing an estimated median ICER of 40,917 (95% CrI: 4,303 to 91,282), SP2 was deemed not cost-effective (cost-effective probability<29%) at the WTP threshold due to additional costs of confirmatory RT-PCR testing and lower QALYs gained. Similar to SP1, SP3 was cost-effective with a 82% probability (at the WTP threshold) and an estimated median ICER of 14,509 (95% CrI: −21,018 to 50,235) per QALY gained. The median iNMB associated with SP1 and SP3 were positive at $1.1 and 2 million, respectively, but with SP2 was negative at $−1.5 million per 100,000 population (Table 3). Similar outcomes were obtained when RA screening was implemented for the entire 1-year simulation timelines, resulting in SP1 and SP3 being cost-effective with probabilities of 72% and 90%, respectively, and SP2 not cost-effective (cost-effectiveness probability<38%) (Fig. 3, Additional file 1: Table S5). In this scenario with 1-year duration of screening, the iNMB obtained from all scenarios did not differ [Mann–Whitney *U* test, *p*>0.5]. When screening started late during a surge (Fig. 2A4, A5), neither RA testing programs were cost-effective at the WTP threshold (Fig. 3), generating negative iNMB compared to the baseline of TOSC (Table 3).

### Cost-effectiveness of testing programs (increased booster coverage)

Increasing booster vaccination coverage among working adults aged 18 to 65 years improved the iNMB of screening programs (Table 3). For a 16-week duration of screening initiated early in a surge within the exponential growth of cases, the per capita iNMB generated by SP1 and SP2 were statistically not different [Kruskal-Wallis test, *p*>0.17], but both were different from SP3 [Kruskal-Wallis test, *p*<0.001]. However, when the screening programs were extended to 32 weeks or 1 year, SP2 generated a greater per capita iNMB [Kruskal-Wallis test, *p*<0.001] than SP1 or SP3. Compared to TOSC, the RA screening programs started late during a surge (Fig. 2B4, B5) were not cost-effective and generated negative iNMB (Table 3).

Increasing booster coverage of working adults by 80% over the status quo resulted in qualitatively similar outcomes (Table 3). However, SP1 and SP2 performed equivalently with similar iNMB [Kruskal-Wallis test, *p*<0.001], but higher than iNMB generated by SP3 for all programs that started early in the surge (Table 3). Neither screening programs were cost-effective and generated negative iNMB when started late during the outbreak. Our results remained qualitatively intact when 100% of workplaces with 50+ employees participated in the screening programs (Additional file 1).

Simulating scenarios with BD Veritor and Sofia RA tests, we found qualitatively similar trends in the cost-effectiveness of the screening program and the iNMB generated by SP1, SP2, and SP3 (Additional file 1: Tables S8-S11). Specifically, screening programs with early start during the exponential growth of a surge were cost-effective, and increasing booster coverage of vaccination improved their iNMB. However, iNMB achieved in each specific screening program varied by the type of RA test, indicating the influence of the test sensitivity on monetary benefits. Cost-effectiveness analyses of the RA screening programs using only direct costs of healthcare and testing (excluding indirect costs) revealed similar outcomes, with a greater iNMB obtained by SP1 than SP2 or SP3 in all simulated scenarios under the same booster vaccination coverage (Additional file 1: Tables S12-S17).

## Discussion

In this study, we evaluated the cost-effectiveness of workplace screening post-Omicron wave of BA.1 variant. We found that increasing booster coverage of working adults improved outcomes and therefore higher net monetary benefits of the screening program would be expected under a higher coverage of booster doses. In addition to booster vaccination, the timing for the start of RA screening during a surge and the duration of the program can have a large impact on the cost-effectiveness of the testing strategy. Delaying the start of RA screening until after the exponential growth or around the peak of a surge would not be a cost-effective strategy. Overall, an RA screening program without a confirmatory test may be a preferred strategy.

Although cost-effectiveness analysis is vital to policy decision-making regarding testing strategies, determining an optimal RA screening program is a challenging task [74, 75]. Previous research on RA testing within schools has highlighted how the optimal testing strategy is dependent on the specific objectives and their associated tradeoffs [76]. These tradeoffs arise from the interplay between testing and incidence: more frequent testing reduces the extent of transmission in the community from the identified cases, which may then justify less frequent testing. Furthermore, the evolving nature of the pandemic may change the cost-effectiveness of workplace-screening programs, including the immune-evasiveness and transmissibility of the virus, levels of vaccine-elicited protection, and durability of immunity against reinfection [77, 78].

Our findings rest on a number of simplifying assumptions in the model. First, we assumed that all individuals who test positive self-isolate for 5 days (SP1) or at least 1 day (SP2, SP3) if the confirmatory RT-PCR or RA test was negative. The implication of this assumption is that their daily contacts, both in and outside the workplace, are substantially reduced. For the screening program with a confirmatory RT-PCR test (SP2), we assumed only a 1-day turnaround time for the results without delay in sample collection from the time of the first positive RA test. However, additional delay in sample collection and results could alter our results further in favor of only RA tests for asymptomatic screening. For the scenarios evaluated here, we considered a frequency of two RA tests per week, as recommended by the stakeholders and consensus among participants in the Health Canada workshop held on January 31, 2022. However, the frequency of testing may vary among different workplaces [79] and could affect the cost-effectiveness results.

Our analysis is based on temporal sensitivity of Abbot-Panbio, BD Veritor, and Sofia rapid antigen tests; however, there are several RA tests currently being used with similar sensitivity and specificity estimates [2]. We calibrated the transmission parameter in the model to the estimated reproduction number in April 2022 [25], which implicitly accounted for the effect of non-pharmaceutical interventions. This transmissibility could change with time (e.g., seasonal effects) and other virus-specific characteristics. For example, with the same transmissibility, the iNMB achieved by implementing a screening program would be expected to decrease for a more severe variant that causes higher rates of hospitalization and/or death. Our analysis was restricted to the size and the proportion of workplaces participating in the screening program without consideration of their type or other attributes [79, 80] such demographics of the workforce, risk of exposure, and contact patterns (e.g., essential workplaces, healthcare facilities or other congregated settings) [7, 81–85]. In settings like hospitals or long-term care facilities, a screening program may consider additional components such as different frequency of RA testing for employees and patients, or screening of visitors. We considered a 10% naturally-acquired immunity at the initiation of the model based on incidence of disease as of April 2022 in Ontario; however, this level is unlikely to alter the qualitative aspect of the results due to a significantly higher level of immunity generated by the two-dose vaccination in primary series.

## Conclusions

Our findings provide important insights which can inform testing strategies. The modeling outcomes suggest that RA testing of asymptomatic infection could provide substantial economic benefits, especially when the capacity for RT-PCR testing with rapid turnaround times is limited. Coverage of booster vaccination among working adults remains an important consideration in the implementation of RA screening. As the booster coverage increases, greater monetary benefits may be achieved by an RA workplace-screening program; however, depending on the starting time of screening during a surge, such benefits may be comparable or even lower than those accrued by testing only severe cases.

## Supplementary Information

**Additional file 1.** Details of the model with its parameterization and additional results.

**Additional file 2.** Details of costs.

## Data Availability

Details of the model are provided in Additional file 1. Computational model for this simulation study is freely available at: https://github.com/thomasvilches/testing_COVID.

## Abbreviations

RT-PCR: Reverse transcription polymerase chain reaction
RA: Rapid antigen
SP: Screening program
TOSC: Testing of only severe symptomatic cases
ICER: Incremental cost-effectiveness ratio
QALY: Quality-adjusted life year
iNMB: Incremental net monetary benefits
