# Supplementary material for: Economic evaluation of COVID-19 rapid antigen screening programs in the workplace
Source: BMC Med. 2022 Nov 23;20:452. doi: 10.1186/s12916-022-02641-5 (PMC9686464; doi:10.1186/s12916-022-02641-5)
Supplement: Supplementary file 1 — Additional file 1. Details of the model with its parameterization and additional results. [file 12916_2022_2641_MOESM1_ESM.docx]

**Supplementary File 1**

**Economic Evaluation of COVID-19 Rapid Antigen Screening Programs in the Workplace**

Thomas N. Vilches^1^, Ellen Rafferty^2^, Chad R. Wells^3^, Alison P. Galvani^3^, Seyed M. Moghadas^1^

^1^ Agent-Based Modelling Laboratory, York University, Toronto, Ontario, Canada

^2^ Institute of Health Economics, Edmonton, Alberta, Canada

^3^ Center for Infectious Disease Modeling and Analysis, Yale School of Public Health, New Haven, Connecticut, USA

This supplement provides an overview of the agent-based simulation model and its parameterization for the derivation of health outcomes used in the cost-effectiveness analysis of rapid antigen screening programs in workplaces.

**Content**

1. Model structure
2. Distribution of disease stages and infectiousness
3. Disease outcomes
4. Vaccine effectiveness and immune dynamics
5. Temporal sensitivity of RT-PCR and RA tests
6. Cost and outcome parameters used in cost-effectiveness analysis
7. Model implementation
8. Additional results
9. Estimated iNMB for the screening programs compared to TOSC with only direct costs of healthcare and testing (excluding indirect costs)
10. **Model structure**

We adapted a previously established agent-based model of COVID-19 transmission (19–21) to account for the waning of naturally acquired or vaccine-elicited immunity. The model implemented natural history of disease with the epidemiological classes of individuals as susceptible; latently infected (not yet infectious); asymptomatic (and infectious); pre-symptomatic (and infectious); symptomatic (and infectious) with either mild or severe illness; recovered; and dead (Figure S1).


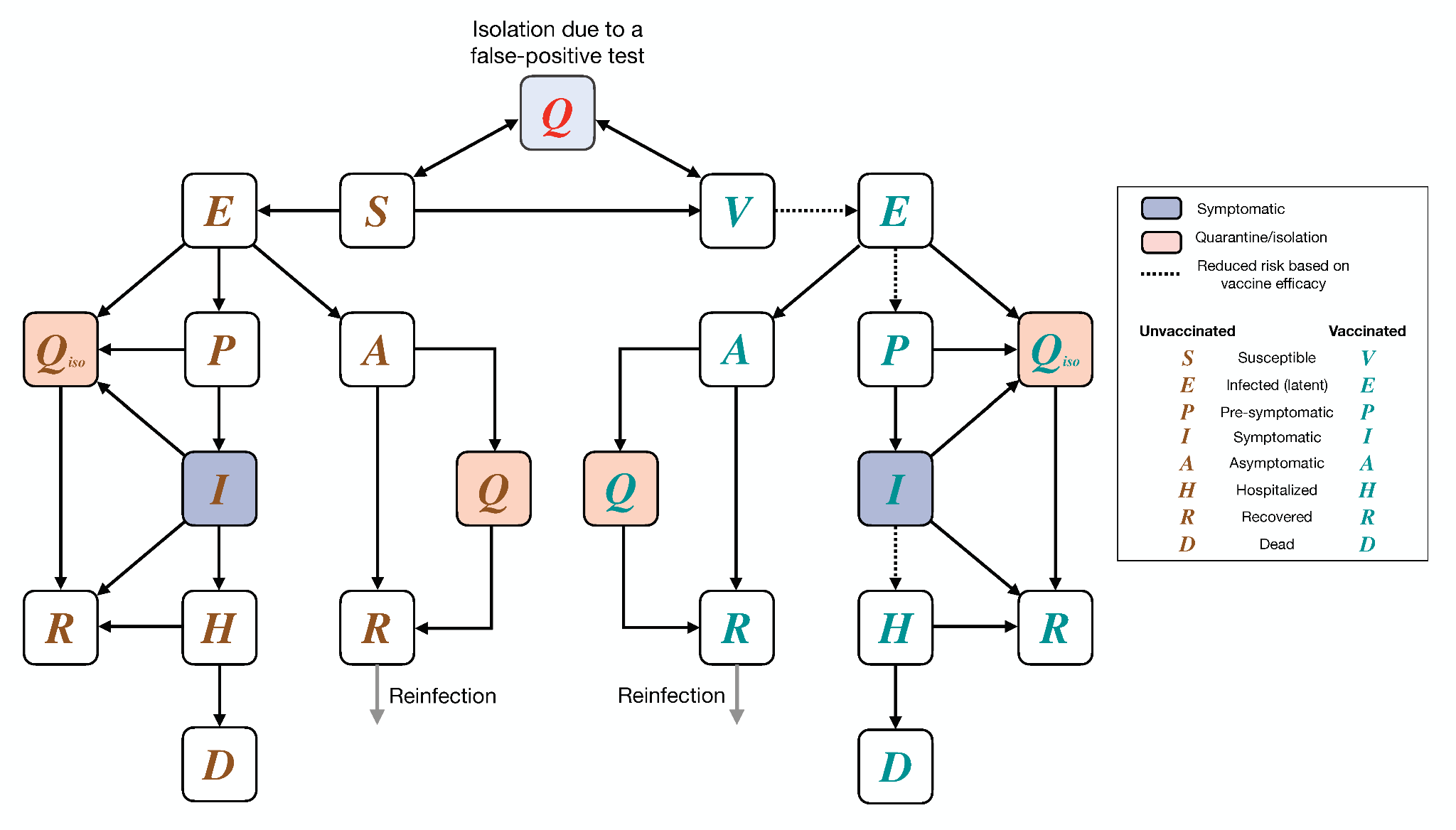


**Figure S1.** Model structure for the dynamics of disease. Q represents isolation of individuals with a positive RT-PCR or RA test.

We stratified the model population into seven age groups of 0 to 4, 5 to 11, 12 to 17, 18 to 49, 50 to 64, 65 to 79, and 80+ years based on demographics of Ontario, Canada (22). To include within and between age-group contacts, we relied on a population-based study of social contacts in Canada (23). Daily contacts for each individual were sampled from a negative binomial distribution with the mean and standard deviation derived from the study (Table S1). We also accounted for the effect of interventions such as isolation of symptomatic individuals on the reduction of daily contacts (Table S1).

**Table S1.** Mixing patterns and the daily number of contacts derived from empirical observations.

| Age group | Proportion of contacts between age groups | | | | | No. of  daily contacts  Mean (SD) | No. of  daily contacts for isolated individuals  Mean (SD) |
| --- | --- | --- | --- | --- | --- | --- | --- |
|  | 0-4 | 5-19 | 20-49 | 50-65 | 65+ |  |  |
| 0-4 | 0.25 | 0.132 | 0.44 | 0.144 | 0.034 | 6.97 (5.22) | 1.95 (1.46) |
| 5-19 | 0.0264 | 0.43 | 0.404 | 0.108 | 0.0316 | 9.54 (6.66) | 2.67 (1.86) |
| 20-49 | 0.03 | 0.13 | 0.602 | 0.179 | 0.059 | 10.96 (8.35) | 3.07 (2.34) |
| 50-65 | 0.026 | 0.086 | 0.456 | 0.3 | 0.132 | 8.05 (6.86) | 2.255 (1.92) |
| 65+ | 0.012 | 0.052 | 0.303 | 0.266 | 0.367 | 4.41 (3.83) | 1.234 (1.12) |

To implement RA screening, we used the distribution of workplace sizes for Ontario (24), the most populated province of Canada.


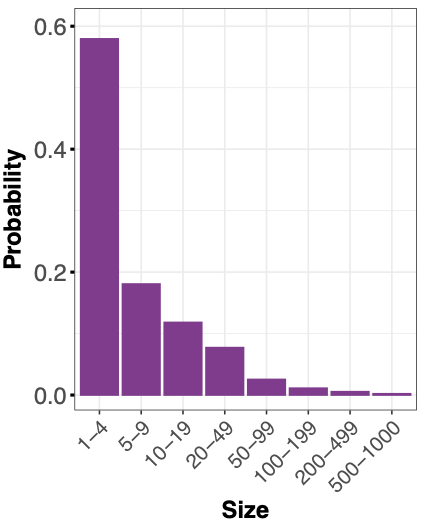


**Figure S2.** Distribution of workplace sizes in Ontario.

1. **Distribution of disease stages and infectiousness**

We considered disease spread with an Omicron subvariant, and calibrated transmission probability to an effective reproduction number of 1.2 in Ontario (25). The incubation period for the Omicron variant is estimated to be shorter than previous variants (26,27). For each infection, we sampled the incubation period from a log-normal distribution with a mean of 3.3 days (26,27). We considered a mean pre-symptomatic stage of 2 days, as part of the incubation period. A proportion of infected individuals progressed to a pre-symptomatic stage (28) with an infectious period which was sampled from a Gamma distribution with a mean of 2 days. The symptomatic disease following the pre-symptomatic stage had an average infectious period of 3.2 days (29,30), which was also sampled from a Gamma distribution. For those who remained asymptomatic throughout the entire infection, the infectious period was sampled from a Gamma distribution with a mean of 5 days (29,31).

Infectiousness was assumed to be highest during the pre-symptomatic stage (32). The transmissibilities during asymptomatic, mild symptomatic, and severe symptomatic stages were 26%, 44%, and 89%, respectively, relative to the pre-symptomatic stage (30,33,34).

1. **Disease outcomes**

We assumed that asymptomatic and mild symptomatic individuals recover without any treatment or need for hospitalization. For individuals who tested positive, self-isolation was implemented for a period of 5 days, reducing their daily number of contacts (Table S1). Severely ill individuals who were hospitalized were effectively excluded from the chain of disease transmission. We considered a 75.2% (95% confidence interval: 72.0% – 77.0%) risk reduction of hospitalization for severe disease due to infection by Omicron compared to Delta (35,36). The risk of ICU admissions was reduced by 38.1% in severe patients of Omicron compared to those infected with Delta (35).

1. **Vaccine effectiveness and immune dynamics**

We performed a literature review to derive the effectiveness estimates following each dose of vaccine against infection, symptomatic disease, and severe disease (Tables S2 S3). We assumed the same degree of reduced protection in naturally-acquired immunity as vaccine-induced immunity (without booster) against Omicron. However, natural immunity was associated with 3.1 times (95% confidence interval: 1.4 – 4.8) lower risk of hospitalization compared to fully vaccinated individuals without a booster and no prior infection (37).

**Table S2.** Estimated vaccine effectiveness (%) and their 95% confidence intervals from published studies for Pfizer-BioNTech vaccines. Booster dose restored or increased the protection efficacy of two doses.

| **Outcome** | **Vaccine effectiveness (%)** | | **Reference** |
| --- | --- | --- | --- |
|  | 1 week after the second dose | 1 week after the booster dose | (38,39) |
| Infection | 33.0  (31.0, 35.0) | 76.0  (72.0, 79.0) |  |
| Symptomatic disease | 69  (62.0, 75.0) | 82.0  (79.0, 84.0) |  |
| Severe disease | 81.0  (65.0, 90.0) | 90.0  (80.0, 94.0) |  |

**Table S3.** Estimated vaccine effectiveness (%) and their 95% confidence intervals against Omicron from published studies for Moderna vaccines. Booster dose restored or increased the protection efficacy of two doses.

| **Outcome** | **Vaccine effectiveness (%)** | | **Reference** |
| --- | --- | --- | --- |
|  | 1 week after the second dose | 1 week after the booster dose | (40,41) |
| Infection | 42.8  (33.8, 50.7) | 67.7  (65.5, 69.7) |  |
| Symptomatic disease | 69  (62.0, 75.0) | 82.0  (79.0, 84.0) |  |
| Severe disease | 81.0  (65.0, 90.0) | 90.0  (80.0, 90.4) |  |

To implement the waning immunity after vaccination, we fitted a Gaussian model to estimates of vaccine effectiveness over time (42-46), and determined the temporal relative effectiveness curves (Figure S3). The relative effectiveness was used as a multiplicative factor in the effectiveness of vaccines after the second dose or booster to determine the temporal immunity of individuals against infection and severe disease. We applied the same relative effectiveness for waning of naturally-acquired immunity.


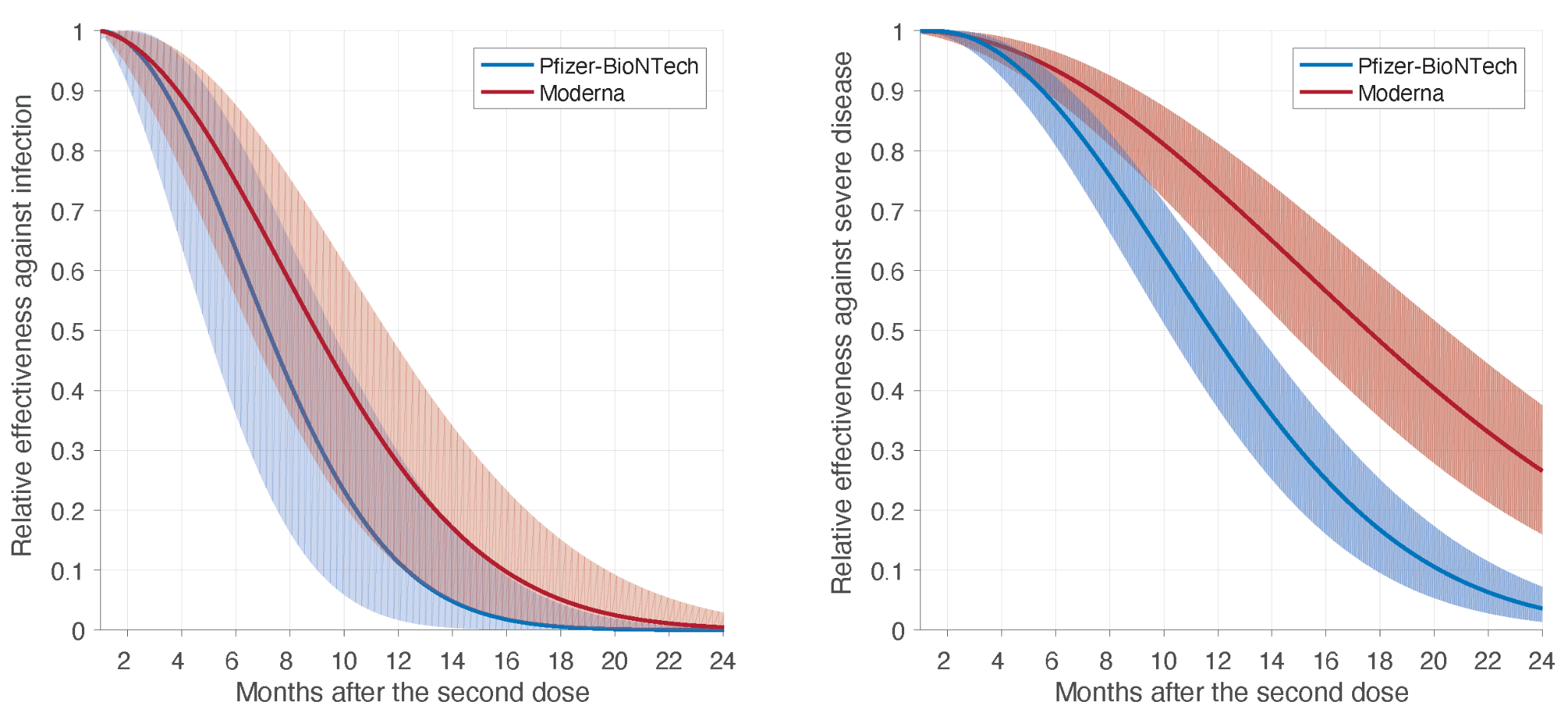


**Figure S3.** Temporal relative effectiveness of vaccines against infection and severe disease derived from Gaussian fit to data of vaccine effectiveness after the second dose.

1. **Temporal sensitivity of RT-PCR and RA tests**

Temporal diagnostic sensitivity was determined for RT-PCR and the Abbot-Panbio, BD Veritor, and Sofia RA test for vaccinated and unvaccinated individuals over the course of disease for incubation periods ranging from one to 28 days long (covering the sampled range from the distribution). These diagnostic sensitivity curves were constructed using a piecewise mapping from the relative infectivity (with the peak infectivity at one) to the diagnostic sensitivity for pre- and post-peak infectivity (6). The relative infectivity profile for each incubation period was constructed using the framework specified by Ferretti et al. (52). For the construction of the mapping from relative infectivity to RT-PCR diagnostic sensitivity, the baseline RT-PCR diagnostic sensitivity curve was estimated for an incubation period of 5.72 days and was inferred by fitting a time-dependent log-Normal probability density function to serial testing data (51) through a maximum likelihood approach (6). This was based on the assumption that the maximum diagnostic sensitivity coincides with the peak of infectiousness, while utilizing the distribution of the incubation period determined previously (53) in the inference of time of infection. Through this baseline mapping for the incubation period of 5.72 days, we determined the diagnostic sensitivity of the RT-PCR test at time *t* post infection for any specified incubation period and vaccine status.

The infectivity profile constructed by Ferretti et al. (52) was based on the ancestral SARS CoV-2 strain for unvaccinated individuals. We used the method specified by Wells et al. (6) to construct the infectivity profile for the Omicron variant of concern, as well as the effects of vaccination on infectivity. The method uses the differences in the linear rate of change in cycle threshold (Ct) values to transform the infectivity profile for the ancestral SARS CoV-2 strain for unvaccinated individuals to the infectivity profile for the Omicron variant of concern (with and without vaccination). These transformed infectivity profiles were used as the inputs in the mapping to determine the RT-PCR diagnostic sensitivity.

To determine the diagnostic sensitivity of the RA test at time *t* post-infection, we computed the product of the RT-PCR diagnostic sensitivity at time *t* and the inferred percent positive agreement of the RA test with RT-PCR at time *t*. The temporal percent positive agreement of the RA test with RT-PCR was determined using a linear logit model. The parameters of the linear logit model were estimated by fitting the model to the percent positive agreement data post-symptom onset for Abbot Panbio (54,55); BD Veritor (56); and Sofia (57) through a maximum likelihood approach (2). To determine the percent positive agreement during the asymptomatic incubation period, we used a similar mapping technique described above based on the relative infectivity (unvaccinated and ancestral strain) post-infectivity peak and the inferred percent positive agreement.

These constructed diagnostic sensitivity curves are continuous in time (i.e., *s*(*t*)). To discretize these diagnostic sensitivities for discrete time values (i.e., *s_t_*), we computed the average diagnostic sensitivity for each specified

$s_{t}=\int_{t-1}^{t} s\left( t \right) dt$

1. **Cost and outcome parameters used in cost-effectiveness analysis**

Table S4 presents the details of various input parameters calculated for the cost-effectiveness analysis.

**Table S4.** Calculations input parameters for cost-effectiveness analysis. All cost estimates are based on Canadian dollars in 2021.

| **Input parameter** | **Calculation** |
| --- | --- |
| RA testing costs | Procurement ($7 per test) + shipping ($1 per test) |
| Emergency room costs | Percent of cases tested positive that went to the emergency room (13.3%) ✕ cost per emergency room visit for COVID-19 ($167) |
| Outpatient costs | Percent of symptomatic people who have an outpatient visit (17.5%) ✕ cost per outpatient visit ($36.9) |
| Chronic outcomes from COVID-19 costs | Likelihood of having chronic outcomes from COVID-19 hospitalization (41.5%) ✕ total cost of chronic COVID-19 outcomes following hospitalization ($50,273) (see additional details below) |
| RT-PCR testing (lost work income) | [Average income per hour in Canada by age group ✕ labour force participation by age group] ✕ [Number of work hours getting PCR (0.5 hours) + number of hours lost waiting for PCR test (7.5 hours)] |
| Positive case (lost work income) | Average income per day in Canada by age group ✕ labour force participation by age group ✕ Number of work hours lost following testing positive (5 days) |
| Hospitalization (lost work income) | Average income per day in Canada by age group ✕ labour force participation by age group ✕ [Length of stay in hospital (12.2 days) + number of days recovering following hospitalization (27 days)] |
| Death due to COVID-19 (lost work income) | See equation below |
| QALY decrement following hospitalization | [Likelihood of having chronic outcomes from COVID-19 hospitalization (41.5%) ✕ total QALY decrement of chronic COVID-19 outcomes following hospitalization (0.78)] + [Length of stay (12.2 days) ✕ In-hospitalization QALY decrement] + additional QALY decrement on discharge. |
| QALY decrement per COVID-19 death | See equation below. |

We calculated the lost productivity from premature death due to COVID-19 using the following formula:


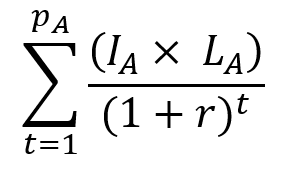


***Where:***

*p_A_* is the number of years lost due to premature death as a function of age.

*t* is the time period, which is determined based on *p_A_*.

*I_A_* is income per year in 2021 as a function of age*.*

*L_A_* is labour force participation in 2021 as a function of age.

*r i*s the discount rate set at 1.5%.

We calculated the QALY decrement form premature death due to COVID-19 using a similar formula:


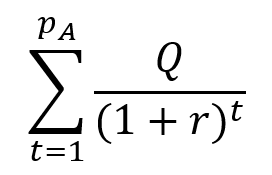


***Where:***

*p_A_* is the number of years lost due to premature death as a function of age.

*t* is the time period, which is determined based on *p_A_*.

*Q* is the Canadian average quality of life per year.

*r i*s the discount rate set at 1.5%.

We estimated the cost and QALY decrement of chronic conditions due to COVID-19 using the technique reported in (66). They estimated QALY decrements and health system costs for six common chronic conditions following hospitalization with COVID-19, including, chronic fatigue, diabetes, chronic kidney disease, chronic liver disease, adverse cardiovascular event, and psychiatric conditions. These conditions were chosen because they had a higher rate following hospitalization for COVID-19, in comparison to controls. We assumed that 41.5% of people hospitalized for COVID-19 ended up with one of the six chronic conditions, which was based on the finding in (67) that over 40% of people discharged from hospitals had persistent symptoms related to chronic illness. To estimate an average cost of chronic outcomes of COVID-19 hospitalization, the six conditions were weighted based on their prevalence in the general population prior to the pandemic. We assumed a 5-year time horizon as a conservative estimate of the long-term impact of COVID-19, and discounted at a rate of 1.5% per year. The results were additionally conservative, because we only assumed chronic outcomes from COVID-19 for those who were hospitalized; however, recent evidence demonstrates that chronic outcomes can occur following infection of all severities.

1. **Model implementation**

With the transmission probability derived from the model calibration to an effective reproduction number of 1.2 with a synthetic population of 100,000 individuals, we simulated screening programs for different timelines. Simulation outcomes were averaged over 500 independent Monte-Carlo realizations, and 95% credible intervals were derived using a bias-corrected and accelerated bootstrap method (with 500 replications).

To ensure an adequate number of Monte-Carlo simulations, we performed an analysis of the coefficient of variation defined by $c_{v}=\sigma/\mu$, where $\sigma$ is the standard deviation of the sample and $\mu$ is its mean. Using an error of $E=0.01$, the minimum number of samples were determined by

$$\text{argmax}_{n}|c_{v}^{x,n}-c_{v}^{x,m} |<E, \text{for} m>n$$

Where $x$ is an outcome. We found that samples of size 500 achieve point of stability for the coefficient of variation. The computational codes for simulations and cost-effectiveness analysis are available at: https://github.com/thomasvilches/testing_COVID.

1. **Additional results**

***8.1. RA test: Abbott-Panbio***

*
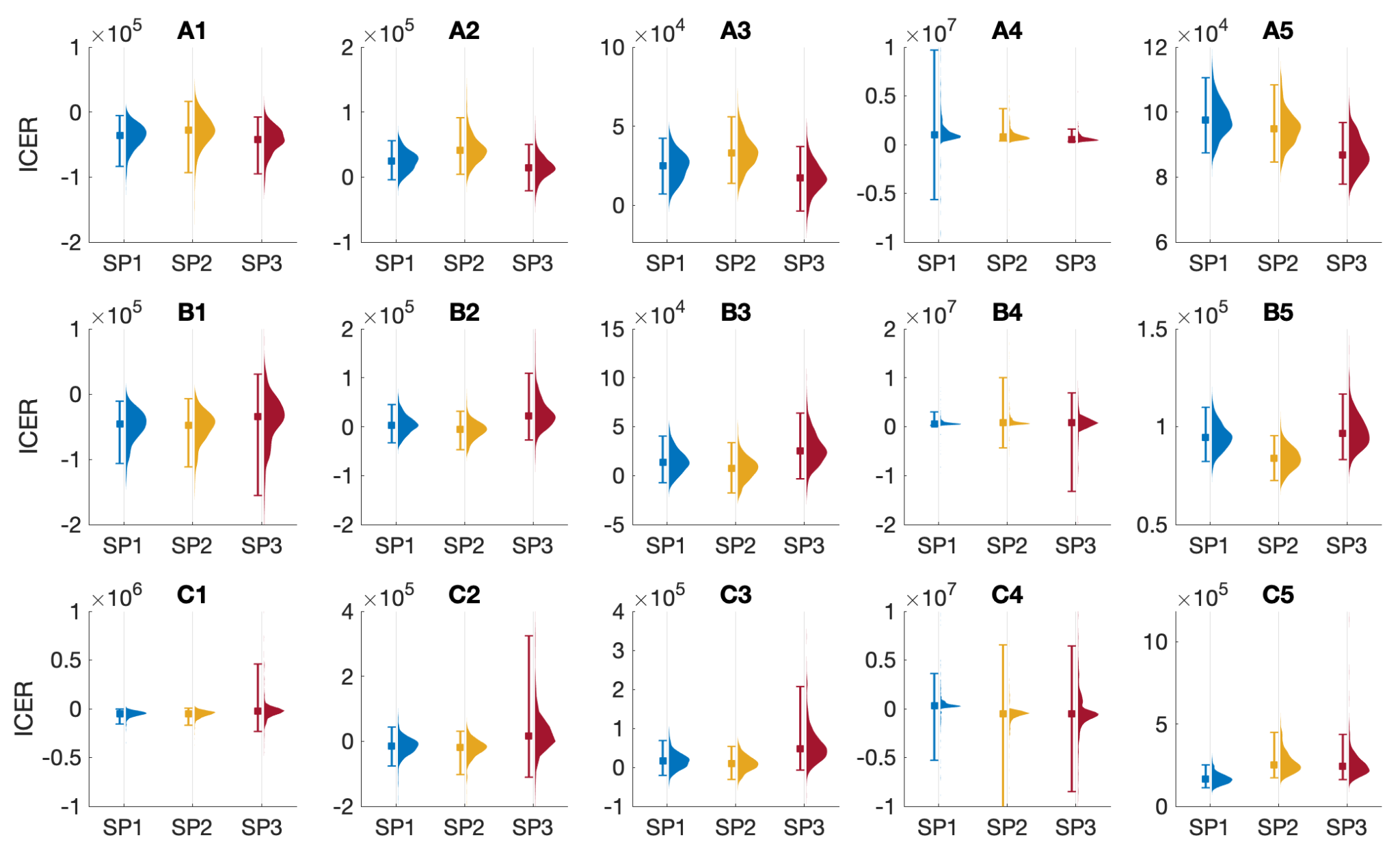
*

**Figure S4.** Distribution of ICER values (reported in Table S4) derived from a bias-corrected and accelerated method using QALYs (as the measure of effects) and costs of the outcomes associated with simulations for different testing scenarios. Screening programs were implemented for 50% of workplaces with a size of 50+ employees. The booster vaccination among adults aged 18-64 years was set to reported coverage as of April 1, 2022 [status quo] (A1-A5); an increase of 20% over status quo (B1-B5); and an increase of 80% over status quo (C1-C5). Comparison was done between the baseline for testing only severe cases (TOSC) and each of the screening programs with the same booster coverage. Bars represent the 95% CrI of the mean ICER values and squares show the median of the distribution.

**Table S5.** Estimated median ICER and 95% credible intervals [CrI] of the mean ICER values for comparing screening programs with the baseline scenario of testing only severe cases, and with timelines corresponding to simulated scenarios in Figure 1 of the main text. The distributions of ICER values are presented in Figure S4.

| **Booster coverage** | **Duration of screening program in weeks** | | | | | | | | | | **Testing program** |
| --- | --- | --- | --- | --- | --- | --- | --- | --- | --- | --- | --- |
|  | **0 to 16** | | **0 to 32** | | **0 to 52** | | **16 to 32** | | **16 to 52** | |  |
|  | ICER | 95% CrI | ICER | 95% CrI | ICER | 95% CrI | ICER | 95% CrI | ICER | 95% CrI |  |
| Status quo as of April 1, 2022 | -35739 | -83556, -5375 | 25033 | -4121, 55925 | 24955 | 7161,  42411 | 983978 | -5643030, 9692643 | 97575 | 87441, 110581 | SP1 |
|  | -27828 | -93085, 16388 | 40917 | 4303,  91282 | 32930 | 13871, 55935 | 780652 | 374081, 3683837 | 94913 | 84626, 108385 | SP2 |
|  | -42166 | -95002, -7681 | 14509 | -21018, 50235 | 17168 | -3602, 37145 | 539637 | 319753, 1589625 | 86718 | 77840, 96782 | SP3 |
| 20% increase over status quo | -46097 | -106465, -10863 | 3078 | -32976, 45267 | 13578 | -7250, 40367 | 615527 | 265025, 3022548 | 94358 | 82211, 109925 | SP1 |
|  | -47433 | -111631, -7104 | -6026 | -46994, 31503 | 7696 | -17729, 33677 | 756030 | -4332779, 10025642 | 83807 | 72486, 95407 | SP2 |
|  | -34989 | -155492, 30552 | 21797 | -27202, 109179 | 109179 | -3281, 63890 | 777913 | -13224172, 6892797 | 96694 | 83183, 116672 | SP3 |
| 80% increase over status quo | -48646 | -155961, -3151 | -14319 | -76038, 43834 | 15887 | -20806, 68534 | 321973 | -5282402, 3614606 | 164213 | 112767, 251645 | SP1 |
|  | -48332 | -168533, 5917 | -18605 | -102344, 30986 | 9545 | -31087, 53442 | -483180 | -12056344, 6556603 | 251754 | 173073, 449254 | SP2 |
|  | -26947 | -232324, 459243 | 16948 | -110390, 324444 | 47967 | -7153, 206953 | -532519 | -8496192, 6443833 | 241606 | 162073, 436689 | SP3 |


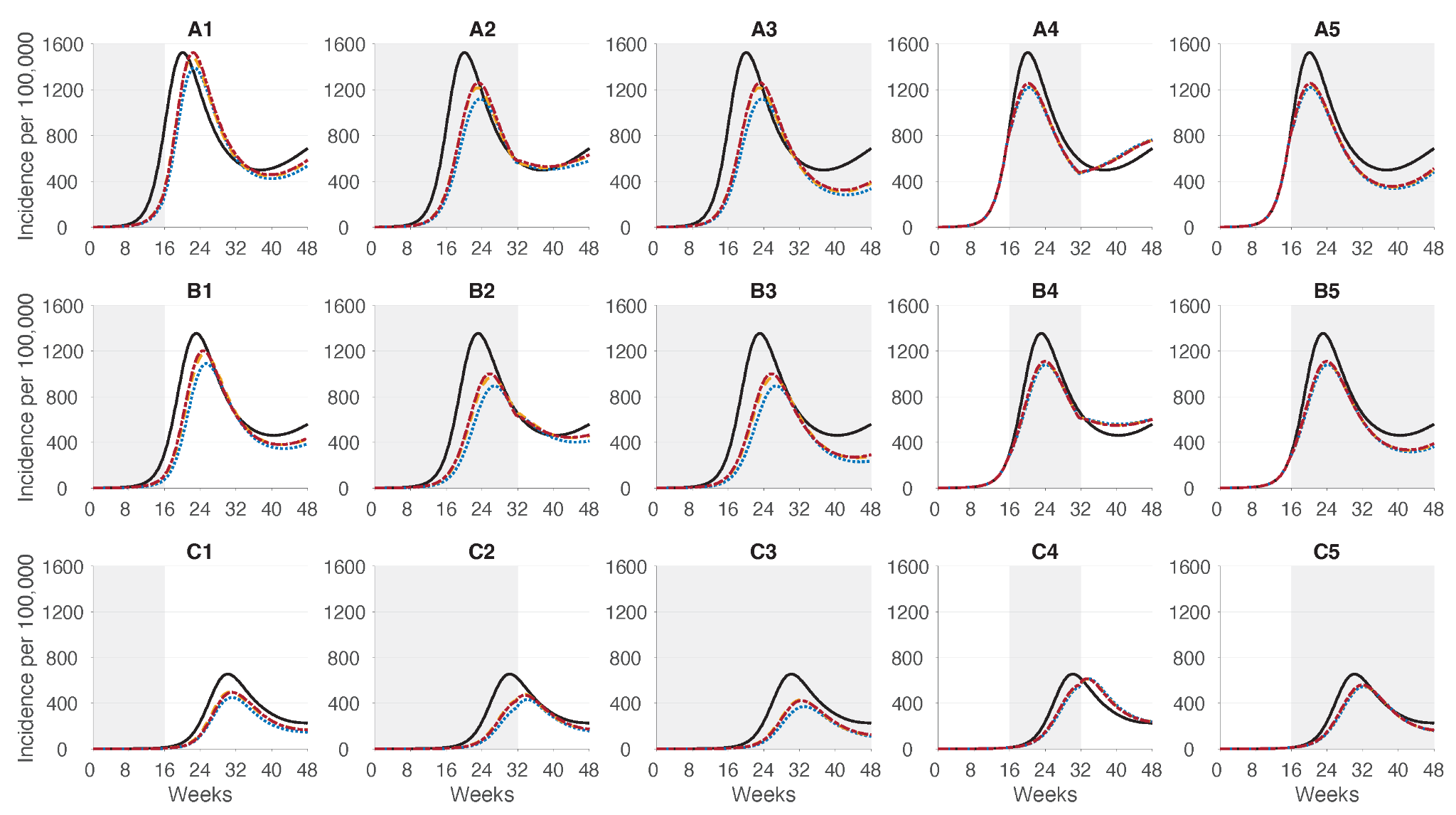


**Figure S5.** Projected average daily incidence of all (symptomatic and asymptomatic) infections for TOSC (black); SP1 in workplaces without a follow-up test (blue); SP2 in workplaces with a confirmatory RT-PCR test (orange); and SP3 in workplaces with a confirmatory RA test (red). Screening programs were implemented for 100% of workplaces with a size of 50+ employees. The booster vaccination among adults aged 18-64 years was set to reported coverage as of April 1, 2022 [status quo] (A1-A5); an increase of 20% over status quo (B1-B5); and an increase of 80% over status quo (C1-C5). Shaded areas indicate the duration of SP1, SP2, and SP3; testing of severe cases with RT-PCR tests was implemented throughout the entire simulation.

*
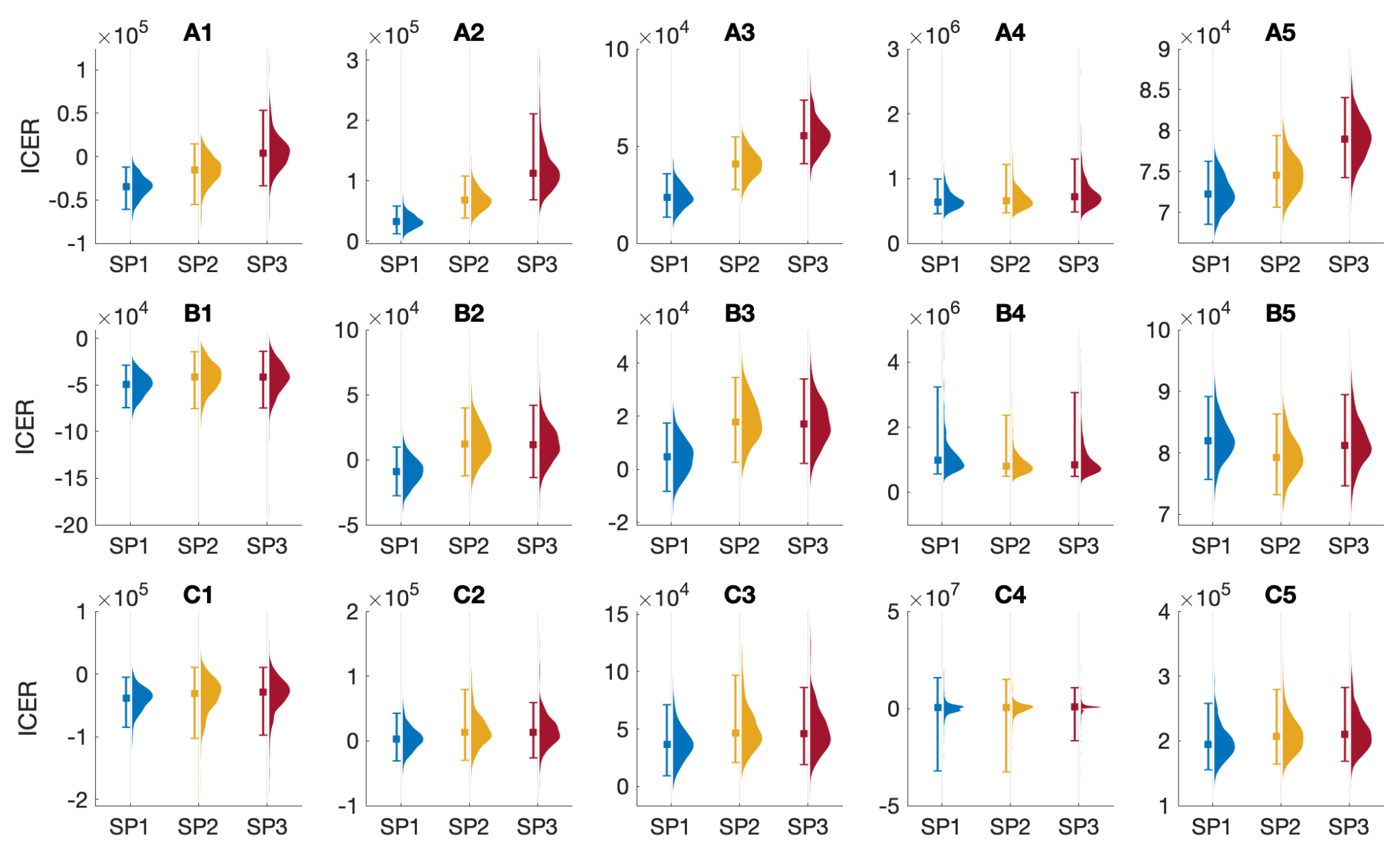
*

**Figure S6.** Distribution of ICER values derived from a bias-corrected and accelerated method using QALYs (as the measure of effects) and costs of the outcomes associated with simulations for different testing scenarios. Screening programs were implemented for 100% of workplaces with a size of 50+ employees. The booster vaccination among adults aged 18-64 years was set to reported coverage as of April 1, 2022 [status quo] (A1-A5); an increase of 20% over status quo (B1-B5); and an increase of 80% over status quo (C1-C5). Comparison was done between the baseline for testing only severe cases (TOSC) and each of the screening programs with the same booster coverage. Bars represent the 95% CrI of the mean ICER values and squares show the median of the distribution.

*
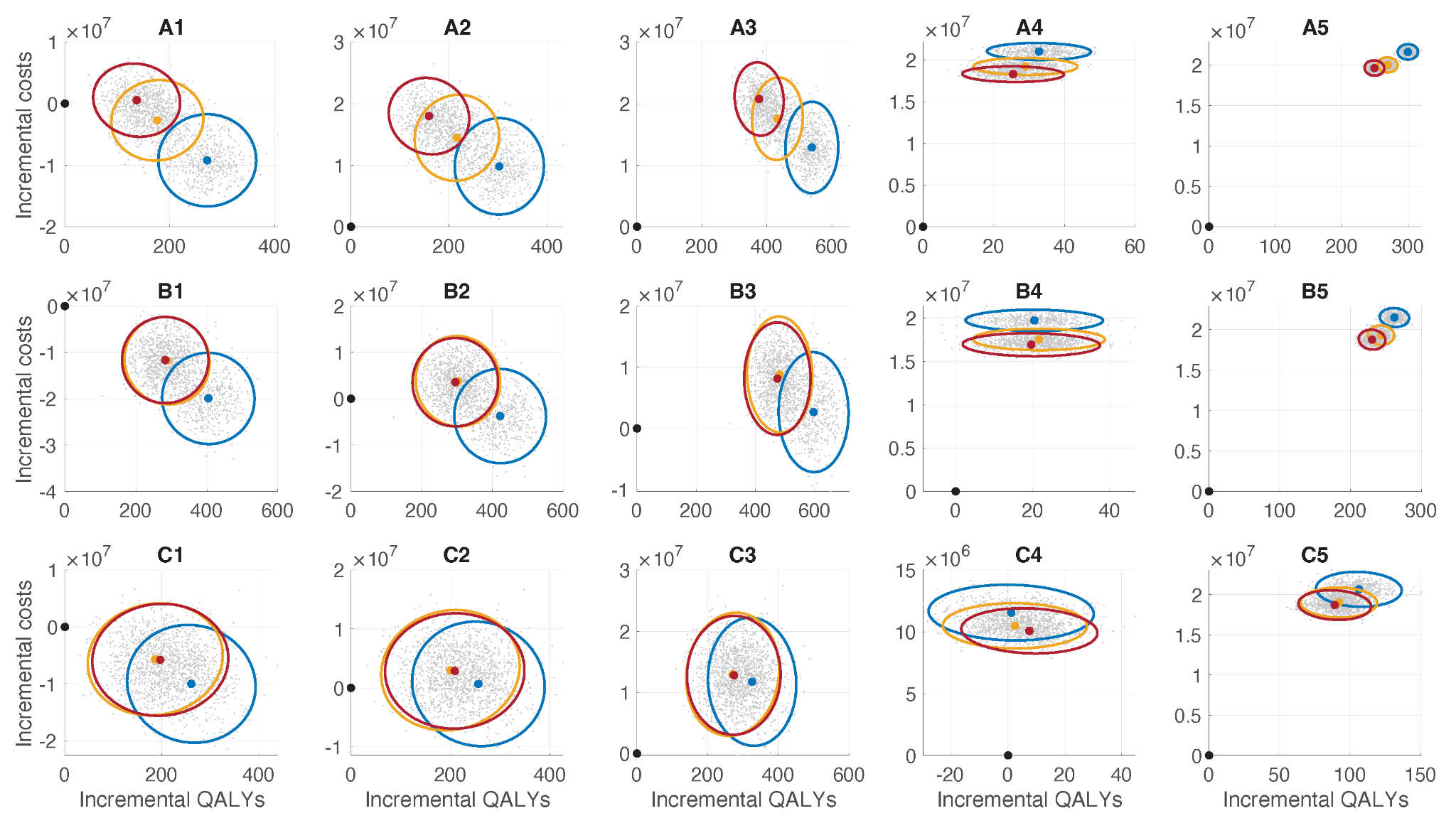
*

**Figure S7.** Cost-effectiveness plane derived from 500 independent Monte-Carlo simulations for different testing scenarios with the associated 95% credible ellipse of the data point distributions. Colors correspond to testing only severe cases (black dot); SP1 in workplaces without a confirmatory test (blue); SP2 in workplaces with a confirmatory RT-PCR test (orange); and SP3 in workplaces with a confirmatory RA test (red). Screening programs were implemented for 100% of workplaces with a size of 50+ employees. The booster vaccination among adults aged 18-64 years was set to reported coverage as of April 1, 2022 [status quo] (A1-A5); an increase of 20% over status quo (B1-B5); and an increase of 80% over status quo (C1-C5). Comparison was done between the baseline for testing only severe cases (TOSC) and each of the screening programs with the same booster coverage. Costs are in Canadian dollars.

**Table S6.** Estimated median ICER and 95% credible intervals [CrI] of the mean ICER values for comparing screening programs with testing only severe cases, and with timelines corresponding to simulated scenarios in Figure S6. The distributions of ICER values are presented in Figure S7.

| **Booster coverage** | **Duration of screening program in weeks** | | | | | | | | | | **Testing program** |
| --- | --- | --- | --- | --- | --- | --- | --- | --- | --- | --- | --- |
|  | **0 to 16** | | **0 to 32** | | **0 to 52** | | **16 to 32** | | **16 to 52** | |  |
|  | ICER | 95% CrI | ICER | 95% CrI | ICER | 95% CrI | ICER | 95% CrI | ICER | 95% CrI |  |
| Status quo as of April 1, 2022 | -34473 | -60827, -12146 | 32378 | 12086, 58071 | 23537 | 13472, 35799 | 639841 | 456802, 994215 | 72197 | 68509, 76232 | SP1 |
|  | -15600 | -55331, 14752 | 67488 | 38132, 107860 | 40742 | 27648, 54747 | 661911 | 469360, 1217720 | 74523 | 70607, 79377 | SP2 |
|  | 4167 | -33707, 53333 | 112899 | 68364, 211128 | 55205 | 40920, 73628 | 721666 | 484482, 1299673 | 78965 | 74230, 84042 | SP3 |
| 20% increase over status quo | -49525 | -74331, -28718 | -8981 | -27568, 9909 | 4718 | -8289, 17471 | 983272 | 564546, 3242341 | 81932 | 75693, 89163 | SP1 |
|  | -41206 | -75268, -14210 | 12149 | -12282, 39910 | 17924 | 2633, 34613 | 814346 | 494406, 2371145 | 79292 | 73220, 86323 | SP2 |
|  | -41392 | -74605, -13803 | 11786 | -13630, 41909 | 17029 | 2230, 34058 | 846658 | 491457, 3067502 | 81206 | 74663, 89475 | SP3 |
| 80% increase over status quo | -37887 | -84774, -5050 | 2536 | -30732, 42662 | 36350 | 9235, 71100 | 442734 | -32107222, 15852436 | 194284 | 155481, 257930 | SP1 |
|  | -30554 | -102518, 10876 | 13364 | -29900, 79393 | 46514 | 20763, 96800 | 684791 | -32581750, 15022768 | 207131 | 164290, 279477 | SP2 |
|  | -29118 | -97258, 10650 | 12818 | -26090, 59237 | 46210 | 19022, 86140 | 747346 | -16564872, 10720793 | 209959 | 168663, 282499 | SP3 |

**Table S7.** Estimated median iNMB (in million CDN$) and 95% credible intervals [CrI] of the mean iNMB values for comparing each testing program with the baseline scenario of testing only severe cases, and with timelines corresponding to scenarios in Figure S6.

| **Booster coverage** | **Duration of screening program in weeks** | | | | | | | | | | **Testing program** |
| --- | --- | --- | --- | --- | --- | --- | --- | --- | --- | --- | --- |
|  | **0 to 16** | | **0 to 32** | | **0 to 52** | | **16 to 32** | | **16 to 52** | |  |
|  | iNMB | 95% CrI | iNMB | 95% CrI | iNMB | 95% CrI | iNMB | 95% CrI | iNMB | 95% CrI |  |
| Status quo as of April 1, 2022 | 17.1 | 10.9, 23.3 | -1.1 | -7.4, 5.2 | 2.9 | -3.2, 8.7 | -20.1 | -20.8, -19.2 | -12.7 | -13.5, -11.9 | SP1 |
|  | 8.1 | 2.0, 13.6 | -8.0 | -14.0, -2.2 | -4.5 | -10.5, 0.9 | -18.3 | -19.2, -17.5 | -11.9 | -12.8, -11.1 | SP2 |
|  | 3.3 | -2.3, 9.2 | -13.4 | -18.9, -7.6 | -9.7 | -15.0, -4.0 | -17.5 | -18.3, -16.6 | -12.2 | -13.0, -11.3 | SP3 |
| 20% increase over status quo | 32.2 | 23.6, 40.1 | 16.6 | 7.3, 24.7 | 15.3 | 6.8, 23.2 | -19.1 | -20.3, -18.0 | -13.6 | -14.7, -12.6 | SP1 |
|  | 20.4 | 12.5, 28.5 | 5.3 | -3.3, 13.4 | 5.6 | -2.4, 13.2 | -16.9 | -18.1, -15.8 | -12.0 | -13.2, -11.0 | SP2 |
|  | 19.9 | 11.3, 27.7 | 5.1 | -3.6, 13.0 | 5.9 | -2.6, 13.4 | -16.3 | -17.5, -15.3 | -11.9 | -13.0, -10.8 | SP3 |
| 80% increase over status quo | 17.9 | 9.6, 26.9 | 7.0 | -1.6, 15.5 | -2.1 | -10.5, 6.6 | -11.7 | -13.2, -9.5 | -17.6 | -19.3, -15.6 | SP1 |
|  | 11.1 | 2.0, 18.6 | 3.0 | -6.7, 10.7 | -4.9 | -14.3, 2.7 | -10.5 | -12.0, -8.8 | -16.3 | -17.9, -14.7 | SP2 |
|  | 11.7 | 4.0, 19.6 | 3.3 | -4.9, 11.5 | -4.8 | -12.6, 3.3 | -9.9 | -11.5, -8.2 | -16.1 | -17.6, -14.4 | SP3 |

***8.2. RA test: BD Veritor***

**Table S8.** Estimated median iNMB (in million CDN$) and 95% credible intervals [CrI] of the mean iNMB values for comparing each testing program with the baseline scenario of testing only severe cases when 50% of workplaces participate in the screening programs.

| **Booster coverage** | **Duration of screening program in weeks** | | | | | | | | | | **Testing program** |
| --- | --- | --- | --- | --- | --- | --- | --- | --- | --- | --- | --- |
|  | **0 to 16** | | **0 to 32** | | **0 to 52** | | **16 to 32** | | **16 to 52** | |  |
|  | iNMB | 95% CrI | iNMB | 95% CrI | iNMB | 95% CrI | iNMB | 95% CrI | iNMB | 95% CrI |  |
| Status quo as of April 1, 2022 | 8.2 | 3.7, 13.8 | -0.7 | -5.2, 5.0 | 1.2 | -3.3, 6.9 | -10.0 | -10.8, -9.1 | -7.6 | -8.5, -6.8 | SP1 |
|  | 6.8 | 2.0, 12.3 | -1.6 | -6.8, 3.9 | -0.1 | -5.1, 5.2 | -10.1 | -10.9, -9.3 | -7.5 | -8.2, -6.8 | SP2 |
|  | 10.0 | 4.7, 15.3 | 1.3 | -4.4, 6.8 | 2.7 | -2.8, 8.0 | -9.2 | -10.2, -8.4 | -6.5 | -7.3, -5.6 | SP3 |
| 20% increase over status quo | 14.8 | 7.4, 22.2 | 6.0 | -2.0, 13.6 | 6.9 | -0.9, 14.1 | -9.7 | -11.0, -8.8 | -7.8 | -8.9, -6.8 | SP1 |
|  | 14.8 | 6.8, 22.2 | 6.3 | -1.6, 13.6 | 6.8 | -1.2, 14.1 | -9.1 | -10.2, -8.1 | -6.7 | -7.8, -5.8 | SP2 |
|  | 7.9 | 0.5, 14.5 | -0.1 | -7.6, 7.0 | 1.0 | -6.4, 8.0 | -8.0 | -9.1, -7.0 | -6.7 | -7.8, -5.8 | SP3 |
| 80% increase over status quo | 10.5 | 1.9, 18.3 | 4.0 | -4.8, 12.1 | -0.3 | -9.2, 7.7 | -4.5 | -6.6, -2.7 | -7.8 | -9.8, -6.1 | SP1 |
|  | 9.3 | 0.8, 18.3 | 5.5 | -3.0, 14.8 | 1.6 | -6.9, 10.8 | -6.8 | -8.0, -5.3 | -9.1 | -10.4, -7.7 | SP2 |
|  | 13.2 | 4.9, 21.8 | 7.0 | -1.9, 15.9 | 3.2 | -5.5, 11.9 | -6.6 | -8.0, -5.1 | -8.9 | -10.3, -7.5 | SP3 |

**Table S9.** Estimated median iNMB (in million CDN$) and 95% credible intervals [CrI] of the mean iNMB values for comparing each testing program with the baseline scenario of testing only severe cases when 100% of workplaces participate in the screening programs.

| **Booster coverage** | **Duration of screening program in weeks** | | | | | | | | | | **Testing program** |
| --- | --- | --- | --- | --- | --- | --- | --- | --- | --- | --- | --- |
|  | **0 to 16** | | **0 to 32** | | **0 to 52** | | **16 to 32** | | **16 to 52** | |  |
|  | iNMB | 95% CrI | iNMB | 95% CrI | iNMB | 95% CrI | iNMB | 95% CrI | iNMB | 95% CrI |  |
| Status quo as of April 1, 2022 | 15.1 | 9.0, 22.2 | -2.9 | -9.2, 4.8 | 2.3 | -3.4, 9.5 | -19.7 | -20.5, -18.9 | -10.6 | -11.4, -9.9 | SP1 |
|  | 9.7 | 4.2, 15.8 | -7.6 | -13.2, -1.0 | -2.9 | -8.3, 3.2 | -18.5 | -19.3, -17.7 | -10.4 | -11.2, -9.6 | SP2 |
|  | 9.8 | 4.3, 16.0 | -7.0 | -12.7, -0.5 | -1.3 | -6.8, 4.9 | -17.8 | -18.7, -17.0 | -10.4 | -11.2, -9.7 | SP3 |
| 20% increase over status quo | 35.9 | 27.3, 43.9 | 19.9 | 11.2, 28.1 | 20.3 | 11.7, 27.6 | -18.8 | -20.0, -17.7 | -11.9 | -13.1, -10.9 | SP1 |
|  | 22.5 | 14.5, 29.9 | 6.9 | -1.4, 14.4 | 7.6 | -0.4, 15.1 | -16.4 | -17.4, -15.3 | -11.3 | -12.4, -10.3 | SP2 |
|  | 20.1 | 11.7, 27.8 | 5.6 | -3.2, 13.8 | 6.4 | -2.1, 14.3 | -15.5 | -16.6, -14.4 | -10.2 | -11.4, -9.1 | SP3 |
| 80% increase over status quo | 20.2 | 12.0, 28.2 | 8.8 | 0.8, 17.3 | -0.3 | -8.2, 8.2 | -11.9 | -13.5, -9.8 | -17.6 | -19.1, -15.7 | SP1 |
|  | 13.3 | 4.1, 21.1 | 3.5 | -5.4, 11.0 | -4.7 | -13.7, 3.0 | -10.1 | -11.5, -8.5 | -16.0 | -17.4, -14.4 | SP2 |
|  | 17.0 | 9.4, 24.7 | 8.0 | -0.1, 15.7 | 0.4 | -7.3, 8.2 | -11.3 | -12.8, -9.8 | -16.2 | -17.6, -14.8 | SP3 |

***8.3. RA test: Sofia***

**Table S10.** Estimated median iNMB (in million CDN$) and 95% credible intervals [CrI] of the mean iNMB values for comparing each testing program with the baseline scenario of testing only severe cases when 50% of workplaces participate in the screening programs.

| **Booster coverage** | **Duration of screening program in weeks** | | | | | | | | | | **Testing program** |
| --- | --- | --- | --- | --- | --- | --- | --- | --- | --- | --- | --- |
|  | **0 to 16** | | **0 to 32** | | **0 to 52** | | **16 to 32** | | **16 to 52** | |  |
|  | iNMB | 95% CrI | iNMB | 95% CrI | iNMB | 95% CrI | iNMB | 95% CrI | iNMB | 95% CrI |  |
| Status quo as of April 1, 2022 | 9.3 | 4.5, 14.6 | -0.5 | -5.3, 5.1 | 1.3 | -3.7, 6.7 | -10.9 | -11.8, -10.1 | -8.8 | -9.7, -8.0 | SP1 |
|  | 9.7 | 4.4, 15.3 | 0.3 | -5.0, 5.9 | 1.7 | -3.7, 7.3 | -9.9 | -10.7, -9.1 | -7.7 | -8.4, -6.8 | SP2 |
|  | 11.5 | 5.7, 17.5 | 2.3 | -3.8, 8.7 | 3.1 | -2.8, 9.3 | -9.0 | -9.9, -8.1 | -7.5 | -8.4, -6.7 | SP3 |
| 20% increase over status quo | 15.4 | 7.6, 22.5 | 6.4 | -1.9, 13.6 | 6.3 | -1.8, 13.4 | -9.0 | -10.2, -8.0 | -8.0 | -9.1, -7.1 | SP1 |
|  | 13.7 | 5.5, 21.5 | 5.3 | -3.2, 13.6 | 5.4 | -2.9, 13.4 | -9.1 | -10.1, -8.2 | -7.4 | -8.5, -6.4 | SP2 |
|  | 8.4 | 0.6, 16.2 | -0.7 | -8.5, 7.3 | -0.8 | -8.4, 7.0 | -8.1 | -9.3, -7.1 | -6.8 | -8.1, -5.8 | SP3 |
| 80% increase over status quo | 14.7 | 7.0, 23.1 | 9.6 | 1.7, 18.1 | 4.6 | -3.3, 13.1 | -5.2 | -6.9, -3.5 | -8.1 | -9.8, -6.2 | SP1 |
|  | 10.9 | 2.0, 19.0 | 6.7 | -2.1, 15.3 | 2.8 | -6.0, 11.5 | -6.5 | -7.7, -5.2 | -9.6 | -11.0, -8.3 | SP2 |
|  | 11.4 | 2.7, 20.3 | 8.6 | -0.3, 17.1 | 4.2 | -4.9, 12.6 | -6.5 | -7.9, -5.0 | -9.2 | -10.4, -7.6 | SP3 |

**Table S11.** Estimated median iNMB (in million CDN$) and 95% credible intervals [CrI] of the mean iNMB values for comparing each testing program with the baseline scenario of testing only severe cases when 100% of workplaces participate in the screening programs.

| **Booster coverage** | **Duration of screening program in weeks** | | | | | | | | | | **Testing program** |
| --- | --- | --- | --- | --- | --- | --- | --- | --- | --- | --- | --- |
|  | **0 to 16** | | **0 to 32** | | **0 to 52** | | **16 to 32** | | **16 to 52** | |  |
|  | iNMB | 95% CrI | iNMB | 95% CrI | iNMB | 95% CrI | iNMB | 95% CrI | iNMB | 95% CrI |  |
| Status quo as of April 1, 2022 | 16.1 | 10.1, 22.9 | -1.3 | -7.7, 6.1 | 3.0 | -3.3, 9.5 | -21.0 | -21.8, -20.2 | -12.4 | -13.1, -11.6 | SP1 |
|  | 8.3 | 2.9, 14.1 | -9.6 | -15.1, -3.6 | -5.5 | -10.8, 0.6 | -19.0 | -19.9, -18.2 | -12.4 | -13.2, -11.7 | SP2 |
|  | 7.7 | 1.7, 13.8 | -10.1 | -16.0, -3.8 | -5.2 | -10.8, 1.1 | -18.2 | -19.0, -17.3 | -11.7 | -12.5, -10.9 | SP3 |
| 20% increase over status quo | 38.0 | 29.3, 46.4 | 21.9 | 13.0, 30.6 | 21.1 | 12.5, 29.6 | -19.2 | -20.4, -18.2 | -13.5 | -14.6, -12.5 | SP1 |
|  | 20.1 | 12.4, 26.9 | 4.6 | -3.3, 11.8 | 5.3 | -2.4, 12.2 | -17.7 | -18.8, -16.6 | -12.6 | -13.8, -11.5 | SP2 |
|  | 19.4 | 11.1, 27.2 | 4.0 | -4.7, 12.1 | 4.3 | -3.8, 12.2 | -16.2 | -17.5, -15.2 | -11.9 | -13.1, -10.9 | SP3 |
| 80% increase over status quo | 21.1 | 13.0, 29.5 | 11.5 | 3.2, 20.3 | 1.9 | -6.1, 10.6 | -11.7 | -13.3, -9.9 | -18.2 | -19.8, -16.3 | SP1 |
|  | 12.1 | 3.3, 20.0 | 3.5 | -5.4, 11.2 | -4.3 | -13.0, 3.3 | -11.0 | -12.5, -9.4 | -16.6 | -18.0, -15.0 | SP2 |
|  | 14.7 | 6.1, 22.7 | 5.4 | -3.7, 13.7 | -2.9 | -11.4, 5.1 | -10.6 | -12.1, -9.1 | -16.0 | -17.5 -14.6 | SP3 |

1. **Estimated iNMB for the screening programs compared to TOSC with only direct costs of healthcare and testing (excluding indirect costs)**

***9.1. RA test: Abbott-Panbio***

**Table S12.** Estimated median iNMB (in million CDN$) and 95% credible intervals [CrI] of the mean iNMB values for comparing each testing program with the baseline scenario of testing only severe cases when 50% of workplaces participate in the screening programs.

| **Booster coverage** | **Duration of screening program in weeks** | | | | | | | | | | **Testing program** |
| --- | --- | --- | --- | --- | --- | --- | --- | --- | --- | --- | --- |
|  | **0 to 16** | | **0 to 32** | | **0 to 52** | | **16 to 32** | | **16 to 52** | |  |
|  | iNMB | 95% CrI | iNMB | 95% CrI | iNMB | 95% CrI | iNMB | 95% CrI | iNMB | 95% CrI |  |
| Status quo as of April 1, 2022 | 7.3 | 4.1, 11.3 | 4.4 | 1.2, 8.4 | 7.2 | 4.0, 11.1 | -3.5 | -4.0, -3.1 | 0.2 | -0.2. 0.7 | SP1 |
|  | 4.2 | 0.5, 7.8 | 0.5 | -3.1, 3.9 | 2.1 | -1.4, 5.7 | -4.0 | -4.5, -3.7 | -1.8 | -2.3, -1.4 | SP2 |
|  | 6.9 | 3.3, 10.8 | 2.9 | -0.6, 7.0 | 4.2 | 0.6, 8.0 | -4.1 | -4.5, -3.7 | -2.2 | -2.5, -1.8 | SP3 |
| 20% increase over status quo | 9.7 | 4.6, 14.8 | 6.3 | 1.1, 11.4 | 8.0 | 3.0, 13.0 | -3.6 | -4.3, -3.0 | -0.5 | -1.2, 0.0 | SP1 |
|  | 9.4 | 4.3, 14.3 | 5.9 | 0.7, 10.9 | 6.1 | 1.0, 11.0 | -4.1 | -4.7, -3.6 | -2.1 | -2.9, -1.6 | SP2 |
|  | 4.6 | -0.4, 9.1 | 1.1 | -3.8, 5.8 | 1.6 | -3.2, 6.3 | -4.4 | -5.1, -3.9 | -3.0 | -3.7, -2.4 | SP3 |
| 80% increase over status quo | 9.4 | 3.9, 14.9 | 5.1 | -0.5, 10.6 | 2.5 | -2.8, 8.0 | -2.9 | -4.1, -1.9 | -4.2 | -5.3, -3.0 | SP1 |
|  | 7.8 | 2.0, 13.4 | 4.2 | -1.6, 10.0 | 1.2 | -4.4, 7.0 | -4.7 | -5.7, -4.0 | -6.5 | -7.4, -5.7 | SP2 |
|  | 3.4 | -1.8, 8.7 | -0.5 | -5.8, 4.8 | -3.3 | -8.5, 1.9 | -4.5 | -5.4, -3.6 | -6.5 | -7.4, -5.5 | SP3 |

**Table S13.** Estimated median iNMB (in million CDN$) and 95% credible intervals [CrI] of the mean iNMB values for comparing each testing program with the baseline scenario of testing only severe cases when 100% of workplaces participate in the screening programs.

| **Booster coverage** | **Duration of screening program in weeks** | | | | | | | | | | **Testing program** |
| --- | --- | --- | --- | --- | --- | --- | --- | --- | --- | --- | --- |
|  | **0 to 16** | | **0 to 32** | | **0 to 52** | | **16 to 32** | | **16 to 52** | |  |
|  | iNMB | 95% CrI | iNMB | 95% CrI | iNMB | 95% CrI | iNMB | 95% CrI | iNMB | 95% CrI |  |
| Status quo as of April 1, 2022 | 12.0 | 7.5, 16.2 | 5.1 | 0.8, 9.3 | 11.2 | 7.2, 14.9 | -6.5 | -6.9, -6.1 | 2.4 | 2.0, 2.9 | SP1 |
|  | 4.4 | 0.6, 8.1 | -3.2 | -7.0, 0.6 | 0.7 | -2.9, 4.2 | -8.4 | -8.8, -8.0 | -2.3 | -2.8, -1.8 | SP2 |
|  | 0.8 | -3.0, 4.6 | -7.3 | -11.0, -3.6 | -3.8 | -7.4, -0.3 | -8.5 | -9.0, -8.1 | -3.5 | -3.9, -3.0 | SP3 |
| 20% increase over status quo | 22.2 | 16.0, 27.6 | 15.0 | 8.7, 20.4 | 16.8 | 11.0, 21.8 | -7.3 | -7.9, -6.7 | 0.0 | -0.8, 0.7 | SP1 |
|  | 13.1 | 7.7, 18.3 | 4.8 | -0.9, 10.1 | 5.8 | 0.4, 10.7 | -8.4 | -9.0, -7.8 | -3.9 | -4.6, -3.3 | SP2 |
|  | 12.4 | 7.1, 17.6 | 4.0 | -1.3, 9.2 | 4.7 | -0.4, 9.7 | -8.7 | -9.4, -8.1 | -4.8 | -5.6, -4.2 | SP3 |
| 80% increase over status quo | 11.4 | 6.0, 17.0 | 3.4 | -2.3, 9.0 | -2.2 | -7.4, 3.2 | -7.6 | -8.8, -6.4 | -10.0 | -11.1, -8.7 | SP1 |
|  | 6.2 | 0.0, 11.3 | -0.7 | -7.0, 4.3 | -7.0 | -13.1, -2.1 | -8.0 | -9.0, -6.9 | -11.9 | -12.9, -10.7 | SP2 |
|  | 6.4 | 0.9, 12.2 | -0.8 | -6.1, 4.9 | -7.4 | -12.3, -1.8 | -7.8 | -8.8, -6.8 | -12.1 | -13.1 -11.0 | SP3 |

***9.2. RA test: BD Veritor***

**Table S14.** Estimated median iNMB (in million CDN$) and 95% credible intervals [CrI] of the mean iNMB values for comparing each testing program with the baseline scenario of testing only severe cases when 50% of workplaces participate in the screening programs.

| **Booster coverage** | **Duration of screening program in weeks** | | | | | | | | | | **Testing program** |
| --- | --- | --- | --- | --- | --- | --- | --- | --- | --- | --- | --- |
|  | **0 to 16** | | **0 to 32** | | **0 to 52** | | **16 to 32** | | **16 to 52** | |  |
|  | **iNMB** | **95% CrI** | **iNMB** | **95% CrI** | **iNMB** | **95% CrI** | **iNMB** | **95% CrI** | **iNMB** | **95% CrI** |  |
| Status quo as of April 1, 2022 | 5.9 | 2.8, 9.8 | 3.1 | 0.1, 7.1 | 6.4 | 3.6, 10.1 | -3.2 | -3.7, -2.8 | 0.8 | 0.4, 1.3 | SP1 |
|  | 4.4 | 0.9, 8.2 | 0.5 | -3.0, 4.2 | 2.7 | -0.6, 6.2 | -4.3 | -4.8, -3.9 | -1.5 | -1.9, -1.1 | SP2 |
|  | 6.6 | 2.9, 10.2 | 2.5 | -1.1, 6.1 | 4.1 | 0.5, 7.7 | -4.3 | -4.7, -3.9 | -1.7 | -2.1, -1.2 | SP3 |
| 20% increase over status quo | 10.2 | 5.3, 15.3 | 6.7 | 1.7, 11.7 | 8.8 | 4.1, 13.7 | -3.6 | -4.3, -3.1 | -0.2 | -1.0, 0.3 | SP1 |
|  | 9.7 | 4.5, 14.5 | 5.3 | 0.2, 10.0 | 6.3 | 1.3, 11.0 | -4.3 | -5.0, -3.8 | -2.0 | -2.7, -1.5 | SP2 |
|  | 4.9 | 0.1, 9.4 | 0.6 | -4.3, 5.3 | 1.8 | -3.0, 6.5 | -4.1 | -4.7, -3.5 | -2.4 | -3.1, -1.8 | SP3 |
| 80% increase over status quo | 7.0 | 1.6, 12.5 | 2.4 | -3.0, 7.9 | 0.2 | -5.1, 5.4 | -2.8 | -4.0, -1.6 | -4.1 | -5.4, -2.8 | SP1 |
|  | 5.8 | 0.1, 11.7 | 2.7 | -3.0, 9.1 | -0.2 | -5.7, 6.0 | -4.8 | -5.7, -3.9 | -6.5 | -7.4, -5.7 | SP2 |
|  | 8.6 | 2.9, 13.9 | 3.7 | -2.1, 8.9 | 0.7 | -4.8, 5.8 | -4.7 | -5.5, -3.8 | -6.5 | -7.4, -5.6 | SP3 |

**Table S15.** Estimated median iNMB (in million CDN$) and 95% credible intervals [CrI] of the mean iNMB values for comparing each testing program with the baseline scenario of testing only severe cases when 100% of workplaces participate in the screening programs.

| **Booster coverage** | **Duration of screening program in weeks** | | | | | | | | | | **Testing program** |
| --- | --- | --- | --- | --- | --- | --- | --- | --- | --- | --- | --- |
|  | **0 to 16** | | **0 to 32** | | **0 to 52** | | **16 to 32** | | **16 to 52** | |  |
|  | **iNMB** | **95% CrI** | **iNMB** | **95% CrI** | **iNMB** | **95% CrI** | **iNMB** | **95% CrI** | **iNMB** | **95% CrI** |  |
| Status quo as of April 1, 2022 | 10.5 | 6.6, 15.2 | 3.6 | -0.6, 8.4 | 10.3 | 6.6, 14.7 | -6.7 | -7.1, -6.2 | 3.3 | 2.9, 3.7 | SP1 |
|  | 5.6 | 1.9, 9.8 | -2.6 | -6.2, 1.6 | 2.0 | -1.5, 5.9 | -8.4 | -8.8, -8.0 | -1.4 | -1.8, -0.9 | SP2 |
|  | 5.8 | 1.7, 9.7 | -2.6 | -6.6, 1.4 | 1.8 | -2.0, 5.5 | -8.4 | -8.9, -8.1 | -2.3 | -2.8, -1.9 | SP3 |
| 20% increase over status quo | 24.8 | 18.8, 30.2 | 17.2 | 11.3, 22.8 | 19.6 | 14.1, 24.6 | -7.2 | -7.9, -6.6 | 0.8 | 0.0, 1.5 | SP1 |
|  | 14.5 | 9.1, 19.2 | 5.8 | 0.4, 10.5 | 7.0 | 1.8, 11.5 | -8.2 | -8.8, -7.6 | -3.3 | -4.0, -2.7 | SP2 |
|  | 12.7 | 7.0, 18.1 | 4.3 | -1.3, 9.8 | 5.4 | 0.0, 10.4 | -8.2 | -8.9, -7.7 | -3.7 | -4.5, -3.1 | SP3 |
| 80% increase over status quo | 13.1 | 7.8, 18.3 | 4.9 | -0.4, 10.3 | -0.9 | -5.9, 4.3 | -7.9 | -9.0, -6.6 | -9.9 | -11.0, -8.7 | SP1 |
|  | 7.6 | 2.0, 12.6 | -0.5 | -6.2, 4.4 | -6.8 | -12.2, -2.0 | -8.0 | -8.9, -7.1 | -11.6 | -12.7, -10.6 | SP2 |
|  | 10.3 | 5.3, 15.4 | 2.6 | -2.6, 7.8 | -3.9 | -8.8, 1.3 | -8.6 | -9.5, -7.7 | -12.2 | -13.2, -11.3 | SP3 |

***9.3. RA test: Sofia***

**Table S16.** Estimated median iNMB (in million CDN$) and 95% credible intervals [CrI] of the mean iNMB values for comparing each testing program with the baseline scenario of testing only severe cases when 50% of workplaces participate in the screening programs.

| **Booster coverage** | **Duration of screening program in weeks** | | | | | | | | | | **Testing program** |
| --- | --- | --- | --- | --- | --- | --- | --- | --- | --- | --- | --- |
|  | **0 to 16** | | **0 to 32** | | **0 to 52** | | **16 to 32** | | **16 to 52** | |  |
|  | **iNMB** | **95% CrI** | **iNMB** | **95% CrI** | **iNMB** | **95% CrI** | **iNMB** | **95% CrI** | **iNMB** | **95% CrI** |  |
| Status quo as of April 1, 2022 | 6.7 | 3.4, 10.4 | 3.7 | 0.5, 7.3 | 7.1 | 4.0, 10.5 | -3.1 | -3.6, -2.7 | 0.8 | 0.4, 1.3 | SP1 |
|  | 6.2 | 2.6, 10.0 | 2.1 | -1.7, 6.0 | 4.0 | 0.5, 7.6 | -4.2 | -4.6, -3.8 | -1.4 | -1.9, -1.0 | SP2 |
|  | 7.8 | 3.9, 12.1 | 3.4 | -0.6, 7.9 | 4.8 | 0.9, 9.1 | -4.1 | -4.5, -3.7 | -1.9 | -2.3, -1.5 | SP3 |
| 20% increase over status quo | 10.3 | 5.3, 15.4 | 6.9 | 1.5, 12.0 | 8.9 | 3.8, 13.7 | -3.1 | -3.8, -2.6 | 0.0 | -0.7, 0.6 | SP1 |
|  | 9.0 | 3.6, 14.4 | 4.8 | -0.6, 10.0 | 5.5 | 0.3, 10.6 | -4.2 | -4.8, -3.7 | -2.1 | -2.8, -1.6 | SP2 |
|  | 4.9 | 0.0, 10.1 | 0.3 | -4.6, 5.6 | 1.1 | -4.0, 6.2 | -4.1 | -4.8, -3.6 | -2.4 | -3.1, -1.8 | SP3 |
| 80% increase over status quo | 10.0 | 4.9, 15.6 | 6.4 | 1.3, 11.8 | 3.8 | -1.2, 9.1 | -3.0 | -4.1, -1.8 | -4.1 | -5.2, -2.9 | SP1 |
|  | 6.9 | 1.1, 12.4 | 3.7 | -2.2, 9.3 | 0.8 | -5.0, 6.3 | -4.7 | -5.7, -3.9 | -6.6 | -7.4, -5.8 | SP2 |
|  | 7.5 | 1.1, 13.1 | 4.7 | -1.5, 10.2 | 1.4 | -4.6, 6.8 | -4.6 | -5.4, -3.6 | -6.5 | -7.4, -5.5 | SP3 |

**Table S17.** Estimated median iNMB (in million CDN$) and 95% credible intervals [CrI] of the mean iNMB values for comparing each testing program with the baseline scenario of testing only severe cases when 100% of workplaces participate in the screening programs.

| **Booster coverage** | **Duration of screening program in weeks** | | | | | | | | | | **Testing program** |
| --- | --- | --- | --- | --- | --- | --- | --- | --- | --- | --- | --- |
|  | **0 to 16** | | **0 to 32** | | **0 to 52** | | **16 to 32** | | **16 to 52** | |  |
|  | **iNMB** | **95% CrI** | **iNMB** | **95% CrI** | **iNMB** | **95% CrI** | **iNMB** | **95% CrI** | **iNMB** | **95% CrI** |  |
| Status quo as of April 1, 2022 | 11.1 | 7.0, 15.7 | 5.1 | 0.9, 9.6 | 11.7 | 7.7, 15.7 | -6.8 | -7.2, -6.4 | 3.1 | 2.7, 3.6 | SP1 |
|  | 4.3 | 0.7, 8.1 | -3.9 | -7.5, -0.1 | 0.5 | -2.8, 3.9 | -8.4 | -8.8, -7.9 | -1.9 | -2.3, -1.5 | SP2 |
|  | 4.2 | 0.1, 8.3 | -4.6 | -8.5, -0.5 | -0.3 | -4.0, 3.5 | -8.5 | -8.9, -8.1 | -2.6 | -3.0, -2.2 | SP3 |
| 20% increase over status quo | 26.7 | 21.0, 32.4 | 19.0 | 13.1, 24.8 | 21.0 | 15.7, 26.4 | -7.1 | -7.8, -6.6 | 0.5 | -0.2, 1.2 | SP1 |
|  | 12.8 | 7.2, 17.4 | 4.2 | -1.4, 8.8 | 5.7 | 0.5, 10.0 | -8.7 | -9.4, -8.1 | -3.7 | -4.5, -3.1 | SP2 |
|  | 12.2 | 6.8, 17.4 | 3.5 | -1.8, 8.9 | 4.5 | -0.5, 9.4 | -8.5 | -9.1, -7.9 | -4.2 | -5.0, -3.6 | SP3 |
| 80% increase over status quo | 13.7 | 8.2, 19.5 | 6.7 | 1.5, 12.3 | 0.7 | -4.3, 6.1 | -7.8 | -8.9, -6.7 | -10.1 | -11.3, -8.9 | SP1 |
|  | 6.9 | 1.0, 11.8 | -0.4 | -6.2, 4.3 | -6.5 | -12.1, -2.0 | -8.3 | -9.2, -7.4 | -11.8 | -12.8, -10.8 | SP2 |
|  | 8.7 | 3.2, 13.9 | 0.7 | -4.9, 6.2 | -5.9 | -11.1, -0.6 | -8.2 | -9.1, -7.3 | -12.0 | -13.1, -11.0 | SP3 |
